# Progression of glucose intolerance and cardiometabolic risk factors over a decade in Chinese women with polycystic ovary syndrome: A case-control study

**DOI:** 10.1371/journal.pmed.1002953

**Published:** 2019-10-25

**Authors:** Noel Yat Hey Ng, Guozhi Jiang, Lai Ping Cheung, Yuying Zhang, Claudia Ha Ting Tam, Andrea On Yan Luk, Jianchao Quan, Eric Siu Him Lau, Tiffany Tse Ling Yau, Michael Ho Ming Chan, Chung Shun Ho, Cadmon King Poo Lim, Risa Ozaki, Jin Huang, Kin Hung Liu, Wing Hung Tam, Daljit Singh Sahota, Winnie Chiu Wing Chu, William Goggins, Jean Woo, Tin Chiu Li, Chun Chung Chow, Juliana Chung Ngor Chan, Ronald Ching Wan Ma

**Affiliations:** 1 Department of Medicine and Therapeutics, The Chinese University of Hong Kong, Hong Kong Special Administrative Region, People’s Republic of China; 2 Hong Kong Institute of Diabetes and Obesity, The Chinese University of Hong Kong, Hong Kong Special Administrative Region, People’s Republic of China; 3 Department of Obstetrics and Gynaecology, The Chinese University of Hong Kong, Hong Kong Special Administrative Region, People’s Republic of China; 4 Li Ka Shing Institute of Health Sciences, The Chinese University of Hong Kong, Hong Kong Special Administrative Region, People’s Republic of China; 5 Division of Health Economics, Policy and Management, Li Ka Shing Faculty of Medicine, The University of Hong Kong, Hong Kong Special Administrative Region, People’s Republic of China; 6 Asia Diabetes Foundation, Hong Kong Special Administrative Region, People’s Republic of China; 7 Department of Chemical Pathology, The Chinese University of Hong Kong, Prince of Wales Hospital, Hong Kong Special Administrative Region, People’s Republic of China; 8 Department of Imaging and Interventional Radiology, The Chinese University of Hong Kong, Hong Kong Special Administrative Region, People’s Republic of China; 9 Division of Biostatistics, The Jockey Club School of Public Health and Primary Care, The Chinese University of Hong Kong, Hong Kong Special Administrative Region, People’s Republic of China; 10 Chinese University of Hong Kong-Shanghai Jiao Tong University Joint Research Centre in Diabetes Genomics and Precision Medicine, Hong Kong Special Administrative Region, People’s Republic of China; University of Manchester, UNITED KINGDOM

## Abstract

**Background:**

Polycystic ovary syndrome (PCOS) is associated with increased metabolic risk, though data on long-term follow-up of cardiometabolic traits are limited. We postulated that Chinese women with PCOS would have higher risk of incident diabetes and cardiometabolic abnormalities than those without PCOS during long-term follow-up.

**Methods and findings:**

One hundred ninety-nine Chinese women with PCOS diagnosed by the Rotterdam criteria and with a mean age of 41.2 years (SD = 6.4) completed a follow-up evaluation after an average of 10.6 ± 1.3 years. Two hundred twenty-five women without PCOS (mean age: 54.1 ± 6.7 years) who underwent baseline and follow-up evaluation over the same period were used for comparison. Progression of glycaemic status of women both with and without PCOS was assessed by using 75-g oral glucose tolerance test (OGTT) screening with the adoption of 2009 American Diabetes Association diagnostic criteria. The frequency of impaired glucose regulation, hypertension, and hyperlipidaemia of women with PCOS at follow-up has increased from 31.7% (95% CI 25.2%–38.1%) to 47.2% (95% CI 40.3%–54.2%), 16.1% (95% CI 11.0%–21.2%) to 34.7% (95% CI 28.1%–41.3%), and 52.3% (95% CI 45.3%–59.2%) to 64.3% (95% CI 57.7%–71.0%), respectively. The cumulative incidence of diabetes mellitus (DM) in follow-up women with PCOS is 26.1% (95% CI 20.0%–32.2%), almost double that in the cohort of women without PCOS (*p* < 0.001). Age-standardised incidence of diabetes among women with PCOS was 22.12 per 1,000 person-years (95% CI 10.86–33.37) compared with the local female population incidence rate of 8.76 per 1,000 person-years (95% CI 8.72–8.80) and 10.09 per 1,000 person-years (95% CI 4.92–15.26, *p* < 0.001) for women without PCOS in our study. Incidence rate for women with PCOS aged 30–39 years was 20.56 per 1,000 person-years (95% CI 12.57–31.87), which is approximately 10-fold higher than that of the age-matched general female population in Hong Kong (1.88 per 1,000 person-years, [95% CI 1.85–1.92]). The incidence rate of type 2 DM (T2DM) of both normal-weight and overweight women with PCOS was around double that of corresponding control groups (normal weight: 8.96 [95% CI 3.92–17.72] versus 4.86 per 1,000 person-years [95% CI 2.13–9.62], *p* > 0.05; overweight/obese: 28.64 [95% CI 19.55–40.60] versus 14.1 per 1,000 person-years [95% CI 8.20–22.76], *p* < 0.05). Logistic regression analysis identified that baseline waist-to-hip ratio (odds ratio [OR] = 1.71 [95% CI 1.08–2.69], *p* < 0.05) and elevated triglyceride (OR = 6.63 [95% CI 1.23–35.69], *p* < 0.05) are associated with the progression to T2DM in PCOS. Limitations of this study include moderate sample size with limited number of incident diabetes during follow-up period and potential selection bias.

**Conclusions:**

High risk of diabetes and increased cardiovascular disease risk factors among Chinese women with PCOS are highlighted in this long-term follow-up study. Diabetes onset was, on average, 10 years earlier among women with PCOS than in women without PCOS.

## Abstract 2

Ronald Ching Wan Ma and colleagues reveal the higher incidence of diabetes and CVD in Chinese women with polycystic ovary syndrome.

## Author summary

**Why was this study done?:**

- Besides reproductive abnormality, it is known that women with polycystic ovary syndrome (PCOS) are at high risk of metabolic complications, such as insulin resistance (IR), impaired glucose tolerance (IGT), and type 2 diabetes mellitus (T2DM).
- The majority of the accessible longitudinal studies regarding the progression of T2DM in women with PCOS are from the United States, Australia, and Europe.
- Previous studies have mainly relied on self-report of diabetes status or linkage through national patient registries, which might underestimate the prevalence and incident rate of T2DM in women with PCOS.

**What did the researchers do and find?:**

- During 2016–2017, we prospectively investigated the progression of glycaemic status among 199 Chinese women with PCOS and compared the data with those of 242 women without PCOS by using oral glucose tolerance test (OGTT) for comprehensive glycaemic status screening.
- We found that the age-standardised incidence rate of T2DM among women with PCOS was around 2.5-fold higher compared with the local female population incidence rate.
- We noted that the incidence rate of T2DM of both normal-weight and overweight women with PCOS was around double that of respective control groups. This is a key controversial area in international literature and differs from data on individuals of European descent.
- Diabetes onset was, on average, 10 years earlier among women with PCOS than in women without PCOS.
- Chinese women with PCOS were at 6-fold higher risk of developing T2DM compared with those without PCOS.
- High waist-to-hip ratio (WHR), elevated triglyceride, and presence of hyperandrogenism are associated with the progression to T2DM in PCOS.

**What do these findings mean?:**

- This study highlighted the risk of diabetes and metabolic abnormalities in women with PCOS.
- The study has shed some light on the potential burden of young-onset diabetes associated with PCOS.
- This work has already been considered in the recent international guidelines, suggesting the clinical relevance of this work. Our data provide useful insights that universal screening should be considered in all Chinese adult women with PCOS.
- Our data suggested WHR, elevated triglyceride, and hyperandrogenism are associated with the progression to T2DM in PCOS, although we were unable to explore relationships with IR. Therefore, further research is clearly warranted.

## Introduction

Polycystic ovary syndrome (PCOS) is underpinned by insulin resistance (IR) and is an important risk factor for type 2 diabetes mellitus (T2DM) [1,2]. The overall prevalence of IR among women with PCOS is between 50% and 75% and is greater in obese women with PCOS [3]. A meta-analysis showed that women with PCOS, compared to women without PCOS, had an approximately 2-fold increase in prevalence of impaired glucose tolerance (IGT), T2DM, and metabolic syndrome (MetS) [4]. Women with PCOS have a 2- to 5-fold increased risk of diabetes [5]. Even among lean women with PCOS, 31% have IGT, and 7.5% have T2DM [6]. The conversion from IGT to T2DM is also accelerated in PCOS [1,7]. Obese women with PCOS (BMI > 30) have approximately 4-fold increased risk of developing IGT or T2DM from normal glucose tolerance (NGT) [7], suggesting high prevalence of obesity in PCOS would further exacerbate IR and T2DM risk. In a recent systematic review and meta-regression, it also highlighted that women with PCOS had an increased prevalence of IGT (odds ratio [OR] = 3.26) and T2DM (OR = 2.87) compared to women without PCOS; and prevalence was higher in women with obesity and doubled among studies using self-report or administrative data for diagnostic T2DM [8].

Despite the body of evidence suggesting women with PCOS have IR and other risk factors for developing T2DM and cardiovascular diseases, there is a scarcity of long-term data, especially from non-European populations. The primary aim of this study was to conduct comprehensive follow-up of a cohort of Chinese women with PCOS in order to address some of the current knowledge gaps. In this long-term prospective study, we aimed to evaluate the rate of progression to glucose intolerance or T2DM, as well as changes in cardiometabolic risk factors, approximately 10 years after the baseline evaluation; to compare the overall incidence rate and age-specific incidence rates of T2DM in women with PCOS against the population incidence rate in Hong Kong; and to identify the independent predictors of T2DM progression in PCOS.

## Materials and methods

### Participants

Between July 2003 and April 2007, 295 Chinese premenopausal women with PCOS from the Prince of Wales Hospital (PWH) PCOS Registry were evaluated as part of a cross-sectional study on prevalence of cardiometabolic risk factors in PCOS [9]. Between 3 January 2016 and 4 December 2017, recruited participants were recalled for detailed clinical and metabolic evaluation at the Diabetes Mellitus & Endocrine Research Centre at the PWH as part of this follow-up study. All women with PCOS fulfilled the diagnostic criteria of PCOS based on the 2003 Rotterdam consensus [10].

Another 292 healthy Chinese premenopausal women without PCOS were recruited from a community-based study of cardiometabolic risk during 2003–2006 [11]. All of them had no clinical or biochemical evidence of hyperandrogenism and have no history of oligomenorrhoea or chronic anovulation. They had undergone the same detailed clinical and metabolic evaluation as women with PCOS, including a standard 75-g oral glucose tolerance test (OGTT) at baseline. All women without PCOS were recalled for follow-up metabolic evaluation, including OGTT, approximately 10 years after the initial visit [12]. Participants with hypothyroidism, prolactinoma, nonclassical adrenal hyperplasia, or Cushing’s syndrome were excluded from the study. The study was approved by the Joint Chinese University of Hong Kong–New Territories East Cluster Clinical Research Ethics Committee. Written informed consent was obtained from all participants who were invited to take part in the study.

### Clinical, anthropometrical, and biochemical assessment

A standard questionnaire was used to document personal, medical, and drug history, etc. Cardiovascular risk factors such as tobacco and alcohol use, family history of diabetes, and reproductive history were also recorded. Signs of clinical androgen excess were noted in women with PCOS by assessing the severity of hirsutism, as well as the frequency and methods for hair removal. Body weight (kg), body height (m), and waist and hip circumferences (cm) were measured. Sitting blood pressure (BP) was measured on the right arm twice after a 5-minute rest using a standard sphygmomanometer; and the mean value was taken.

Medical records of women with known onset of T2DM prior to baseline or follow-up assessment were retrieved from the Hospital Authority’s electronic health records system in PWH in order to capture the age at T2DM diagnosis. For women who were newly diagnosed with T2DM on the follow-up visit day, their age of T2DM onset was defined as their age at follow-up.

Overnight-fasting blood specimens were obtained in all women for measurements of fasting glucose (FG), total cholesterol (TC), triglycerides (TGs), high-density lipoprotein cholesterol (HDL-C), and low-density lipoprotein cholesterol (LDL-C). All women with PCOS and women without PCOS underwent a standard 75-g OGTT unless they had a known diagnosis of T2DM or had recently completed OGTT. Fasting insulin, female sex hormones, serum total testosterone, androstenedione, sex hormone–binding globulin (SHBG), and anti-Müllerian hormone (AMH) were measured.

### Laboratory analyses

Plasma glucose, TC, TGs, HDL-C, and LDL-C were measured by appropriate enzymatic methods on the DP Modular Analytics (Roche Diagnostics). Fasting insulin and SHBG were measured using Immulite 1000 Analyzer (Siemens Healthcare Diagnostics). Serum LH, FSH, total estradiol, and total testosterone levels were measured by electro-chemiluminescent immunoassays on the Roche E170 system (Roche Diagnostics). Serum total testosterone at follow-up was measured by the liquid chromatography tandem mass spectrometry (LC-MS/MS) method (Waters Quattro Micro) [13]. Based on the results of 170 archived samples from women with PCOS measured using both platforms and on the Passing and Bablok regression analysis conducted [13], a conversion calculation was performed on the follow-up testosterone levels to facilitate a comparison between the baseline and follow-up total testosterone results. AMH was measured using Elecsys AMH electro-chemiluminescent immunoassay Roche Cobas E411 analyzer (Roche Diagnostics). These measurements were performed using standard reagent kits supplied by the manufacturers. The analytical performance of these assays was within the specifications of the analysers.

### Main outcome measures

Based on the result of a standard 75-g OGTT, dysglycaemia was defined according to the 2009 American Diabetes Association diagnostic criteria [14]. These included impaired fasting glucose (IFG) (FG = 5.6–6.9 mmol/l and 2-hour glucose < 7.8 mmol/l), IGT (FG < 5.6 mmol/l and 2-hour glucose = 7.8–11.0 mmol/l), combined IFG/IGT (FG = 5.6–6.9 mmol/l and 2-hour glucose = 7.8–11.0 mmol/l), and T2DM (FG ≥ 7 mmol/l or 2-hour glucose ≥ 11.1 mmol/l or treatment with antidiabetic medications). Hypertension was defined based on the BP treatment thresholds proposed in the report from the Eighth Joint National Committee as SBP ≥ 140 mmHg or DBP ≥ 90 mmHg or treatment with antihypertensive medication [15]. Dyslipidaemia is defined by TC ≥ 5.2 mmol/l, TG ≥ 1.7 mmol/l, HDL-C <1.3 mmol/l, LDL-C ≥ 3.4 mmol/l, or treatment with lipid-lowering drugs. Hyperandrogenism was defined by the presence of biochemical hyperandrogenism (defined as elevated total testosterone ≥ 3 nmol/l measured by electro-chemiluminescent immunoassay, the latter being the upper limit of our laboratory range for healthy women) and/or clinical hyperandrogenism (defined as mild/moderate/severe hirsutism and/or mild/moderate/severe acne). Free androgen index (FAI) was calculated by 100 × (total testosterone [nmol/l]/SHBG [nmol/l]). Homeostatic model assessment of IR (HOMA-IR) was calculated by (FG [mmol/l] × fasting insulin [mIU/ml])/22.5. HOMA-β was calculated by (20 × fasting insulin [mIU/ml])/(FG [mmol/l] − 3.5).

### Statistical analysis

Statistical analyses were performed using IBM SPSS Statistics Version 24. The distribution of variables was examined using the skewness and kurtosis test. Data with skewness between −1 and 1 were considered as normally distributed. Covariates were categorised or log transformed if needed. Differences between groups were evaluated using independent-sample *t* test for normal distributed data or Mann-Whitney *U* test for skewed data as appropriate for continuous variables. When comparing baseline characteristics between women with or without PCOS, all analyses were adjusted for the differences in age by using either a general linear model for continuous data or multinomial logistic regression for categorical data. To examine differences in frequencies between groups, chi-squared (*X*^2^) or Fisher’s exact tests were used as appropriate for categorical variables. The paired *t* test or the Wilcoxon signed rank test was used to compare continuous variables between the baseline and follow-up visit. The crude incidence rate of T2DM in women with PCOS was computed and expressed as the number of cases of T2DM per 1,000 person-years. Annual incidence rate was adjusted for age by direct standardisation using the Hong Kong mid-year population in 2014 as the standard population. We then compared the crude and age-standardised incidence rate of diabetes against the background population incidence rate of diabetes in Hong Kong, estimated from the health records of 6.4 million Hong Kong residents from 2006 to 2014 [16]. A baseline univariable comparison between DM converters and non-DM converters among women with PCOS was conducted in order to select a group of potential predictors for diabetes in these women, and these subsets of variables were included into the multivariable logistic regression analyses with the aim of identifying independent predictors for T2DM in women with PCOS. In addition, we surveyed some well-known literature to identify other important risk factors for the progression of T2DM in women with PCOS, which were also included serially into our regression models in order to explore their mediating effects on the risk of T2DM. Therefore, the multivariable regression models used in our study were based on prior knowledge and literature, as well as supported by our own data. Covariates were categorised or log transformed for skewed data. Waist-to-hip ratio (WHR) itself resulted in a larger coefficient in logistic regression; therefore, in order to ensure it is on a comparable scale, WHR *z*-score was computed. Other continuous variables were transformed into *z*-scores in order to make direct comparisons. Statistical significance was defined as a two-sided *p*-value < 0.05.

## Results

### Baseline characteristics of recruited participants

Between 3 January 2016 and 4 December 2017, a total of 199 out of 295 (67.5%) women were successfully contacted and recruited for this prospective follow-up study, whereas 96 women were not included in the follow-up because of death (*n* = 1), refusal (*n* = 91), or failure to be contacted (*n* = 4). A total of 166 out of 199 (83.4%) completed an OGTT, whereas 33 (16.6%) did not because of refusal (*n* = 1) or preexisting T2DM (*n* = 32). A total of 242 out of 292 women without PCOS were evaluated using the same follow-up assessment as women with PCOS. A total of 50 healthy women without PCOS who participated in the baseline assessment were excluded because of failure to be contacted, were >55 years old, or had an unknown glycaemic status. For women without PCOS, 185 out of 242 (76.4%) completed an OGTT.

Comparison of baseline characteristics between women with PCOS (mean follow-up time: 10.6 ± 1.3 years) and control participants (mean follow-up time: 11.5 ± 0.6 years) was summarised in Table 1. Compared with women without PCOS, women with PCOS were younger and more obese. Women with PCOS had significantly worse metabolic profiles including higher FG, fasting insulin, HOMA-IR, and fasting TG. Of note, 11.8% of women with PCOS had total testosterone ≥ 3 nmol/l, the upper limit of the reference range. Women with PCOS have significantly higher levels of AMH compared with women without PCOS. All results were adjusted for age at baseline.

**Table 1:** Comparison of baseline and follow-up clinical, anthropometric, and biochemical characteristics in women with or without PCOS.

	PCOS *n* = 199			Control *n* = 242						
Characteristics	(A) Baseline	(B) Follow-up	Mean change ± SD (follow-up − baseline)	(C) Baseline	(D) Follow-up	Mean change ± SD (follow-up − baseline)	*p*-Value A versus C	*p*-Value A versus B	*p*-Value C versus D	*β* coefficient (95% CI) between PCOS and control#, adjusted by age^f^
**Follow-up duration (years)**	10.6 ± 1.3			11.5 ± 0.6			<0.001^a^			
**Age (years)**	30.6 ± 6.5	41.2 ± 6.4	10.7 ± 1.3	42.6 ± 7.0	54.1 ± 6.8	11.5 ± 1.5	<0.001^a^	<0.001^b^	<0.001^b^	−0.79 (−1.07 to −0.52
**BMI (kg/m**^**2**^**)**	25.9 ± 5.6	26.9 ± 5.8	1.1 ± 2.8	23.2 ± 3.8	23.9 ± 4.1	0.9 ± 2.2	<0.001*	<0.001^b^	<0.001^b^	0.14 (−0.38 to 0.67)
**Waist circumference (cm)**	82.6 ± 12.7	84.0 ± 13.9	1.1 ± 7.3	74.8 ± 8.8	80.8 ± 9.7	7.0 ± 6.4	<0.001*	0.05^b^	<0.001^b^	−5.66 (−7.15 to −4.17)
**Waist-to-hip ratio**	0.8 ± 0.1	0.8 ± 0.1	0.03 ± 0.10	0.8 ± 0.1	0.9 ± 0.1	0.1 ± 0.1	<0.001*	<0.001^b^	<0.001^b^	−0.03 (−0.05 to −0.01)
**Systolic BP (mmHg)**	116.9 ± 17.0	120.1 ± 14.8	3.1 ± 14.2	112.4 ± 15.8	121.8 ± 15.0	11.8 ± 13.7	<0.001*	<0.001^b^	<0.001^b^	−9.04 (−11.96 to −6.12)
**Diastolic BP (mmHg)**	71.4 ± 10.8	78.5 ± 12.0	7.0 ± 11.26	72.4 ± 10.0	74.6 ± 9.8	3.6 ± 9.2	0.565*	<0.001^b^	<0.001^b^	3.80 (1.65–5.95)
**Fasting glucose (mmol/L)**	5.2 ± 1.1	5.6 ± 2.1	0.4 ± 1.8	4.9 ± 0.6	5.0 ± 0.5	0.2 ± 0.5	<0.001*	<0.001^b^	<0.001^b^	0.25 (−0.03 to 0.53)
**2-hour glucose (mmol/L)**	7.2± 2.7	7.6 ± 3.3	1.1 ± 2.8	6.0 ± 1.7	6.4 ± 1.9	0.4 ± 1.7	0.018*	<0.001^b^	0.003^b^	0.70 (0.15–1.24)
**Fasting insulin (μU/mL)**	11.4 (6.2–23.2)	8.8 (5.1–13.6)	−2.9 ± 11.5	7.6 (5.0–11.1)	6.69 (4.8–10.8)	−0.4 ± 10.7	<0.001*†	<0.001^d^†	0.278^d^†	−2.45 (−5.06 to 0.16)
**HOMA-IR**	2.8 (1.4–5.5)	2.1 (1.0–3.6)	−0.4 ± 3.5	1.6 (1.0–2.5)	1.5 (1.0–2.3)	−0.02 ± 2.46	<0.001*†	0.009^d^†	0.802^d^†	−0.39 (−1.08 to 0.31)
**HOMA-β**	143.6 (93.8–276.0)	114.4 (73.1–173.2)	−51.0 ± 133.56	125.3 (77.4–192.4)	92.3 (66.3–145.3)	−41.8 ± 171.6	0.535*†	<0.001^d^†	<0.001^d^†	−9.71 (−46.79 to 27.37)
**Total cholesterol (mmol/L)**	4.9 ± 1.0	5.0 ± 0.9	0.1 ± 0.9	5.1 ± 0.9	5.4 ± 0.8	0.4 ± 0.8	0.973*	0.12^b^	<0.001^b^	−0.32 (−0.49 to −0.14)
**HDL-C (mmol/L)**	1.6 ± 0.5	1.5 ± 0.5	−0.1 ± 0.5	1.7 ± 0.5	1.8 ± 0.4	0.02 ± 0.40	0.224*	0.06^b^	0.750^b^	−0.09 (−0.19 to 0.01)
**Triglyceride (mmol/L)**	1.0 (0.7–1.7)	1.1 (0.8–1.7)	−0.03 ± 1.10	0.8 (0.6–1.2)	1.0 (0.7–1.4)	0.2 ± 0.5	<0.001*†	0.165^d^†	<0.001^d^†	−0.26 (−0.44 to −0.08)
**LDL-C (mmol/L)**	2.8 ± 2.0	2.8 ± 0.8	0.02 ± 1.97	2.9 ± 0.9	3.1 ± 0.8	0.3 ± 0.7	0.739*	0.87^b^	<0.001^b^	−0.33 (−0.65 to −0.02)
**FSH (IU/L)**	5.8 ± 1.9	8.5 ± 8.2	2.7 ± 8.0	-	-	-	-	<0.001^b^	-	-
**LH (IU/L)**	9.0 ± 6.6	8.9 ± 8.9	−0.1 ± 11.3	-	-	-	-	0.89^b^	-	-
**Oestrogen (pmol/L)**	138.0 (109.0–208.0)	140.5 (86.3–280.5)	48.2 ± 427.2	-	-	-	-	0.92^b^†	-	-
**Testosterone (nmol/L)**	1.4 (1.1–1.9)	0.8 (0.6–1.2)	−0.5 ± 0.7	-	0.6 (0.4–0.8)	-	-	<0.001^d^†	-	-
**FAI**	4.3 (2.4–7.1)	2.2 (1.5–3.7)	−3.0 ± 6.5	-	-	-	-	<0.001^d^†	-	-
**Androstenedione (nmol/l)**	-	3.7 ± 2.0	-	-	-	-	-	-	-	-
**17-OH progesterone (nmol/l)**	-	1.6 ± 1.7	-	-	-	-	-	-	-	-
**AMH (pmol/L)**	27.1 (19.1–44.8)	12.6 (4.7–29.7)	−13.5 ± 27.9	1.8 (0.1–7.6)	-	-	<0.001^e^§	<0.001^d^§	-	-
**Family history of T2DM^**	-	51.8% (44.8%–58.7%)	-	-	40.5% (34.3%–46.7%)	-	-	-	-	-
**Use of metformin**	12.6% (8.0%–17.2%)	30.2% (23.8%–36.5%)	17.6%	0.4% (−0.4 to 1.2)	-	-	<0.001^c^	<0.001^c^	-	-
**Use of antihypertensive medications**	3.5% (1.0%–6.1%)	21.1% (15.4%–26.8%)	17.6%	2.5% (0.5%–4.4%)	-	-	0.521^c^	<0.001^c^	-	-
**Use of lipid-lowering drugs**	0.5% (−0.5% to 1.5%)	17.6% (12.3%–22.9%)	17.1%	-	-	-	-	<0.001^c^	-	-
**IGR**	31.7% (25.2%–38.1%)	47.2% (40.3%–54.2%)	15.5%	23.6% (18.2%–28.9%)	33.5% (27.5%–39.4%)	9.9%	0.004^c^	0.002^c^	0.02^c^	-
**DM**	9.5% (5.5%–13.6%)	26.1% (20.0%–32.2%)	16.6%	7.0% (3.8%–10.2%)	17.1% (12.2%–22.0%)	10.1	0.028^c^	<0.001^c^	<0.001^c^	-
**DM among women with BMI < 23**	0%	9.2% (2.7%–15.7%)	9.2%	4.5% (1.0%–8.0%)	10.8% (5.1%–15.9%)	6.3%	-	-	0.067^c^	-
**DM among women with BMI ≥ 23**	15.4% (9.1%–21.8%)	36.6% (28.1%–45.1%)	21.2%	10.1% (4.4%–15.7%)	25% (16.7%–33.3%)	14.9%	0.037^c^	<0.001^c^	0.004^c^	-
**HT**	16.1% (11.0%–21.2%)	34.7% (28.1%–41.3%)	18.6%	10.3% (6.5%–14.2%)	4.3% (1.4%–7.2%)	−6%	0.026^c^	<0.001^c^	0.02^c^	-
**Dyslipidaemia**	52.3% (45.3%–59.2%)	64.3% (57.7%–71.0%)	12%	69.8% (64.1%–75.6%)	65.4% (58.6%–72.2%)	−4.4%	0.254^c^	0.015^c^	0.33^c^	-

Data were presented as means ± SD, proportion in % (95% CI), or median (interquartile range) and analysed by the following, as appropriate:

^a^Independent-sample *t* test.

^b^Paired-samples *t* test.

^c^*X*^2^/Fisher’s exact tests.

^d^Wilcoxon signed rank test.

^e^Mann-Whitney *U* test.

^f^Linear regression.

*Adjusted for the differences in age by using either a general linear model for continuous data or multinomial logistic regression for categorical data.

#Control cohort was used as the reference group.

†Log transformation was used when being analysed.

§Inverse normal transformation was used when being analysed.

^Family history of T2DM was defined as either parent having a diagnosis of T2DM. HOMA-IR was calculated by (fasting glucose [mmol/l] × fasting insulin [mIU/ml])/22.5. HOMA-β was calculated by (20 × fasting insulin [mIU/ml])/(fasting glucose [mmol/l] − 3.5). FAI was calculated by 100 × (total testosterone [nmol/L]/sex hormone–binding globulin [nmol/L]). IGR includes IFG, IGT, IFG/IGT, and DM.

Abbreviations: AMH, anti-Müllerian hormone; BP, blood pressure; DM, diabetes mellitus; FAI, free androgen index; FSH, follicle-stimulating hormone; HDL-C, high-density lipoprotein cholesterol; HOMA-β, homeostasis model assessment of beta cell function; HOMA-IR, homeostasis model assessment of insulin resistance; HT, hypertension; IFG, impaired fasting glucose; IGR, impaired glucose regulation; IGT, impaired glucose tolerance; LDL-C, low-density lipoprotein cholesterol; LH, luteinizing hormone; PCOS, polycystic ovary syndrome; T2DM, type 2 DM

In order to evaluate any potential selection bias, baseline characteristics of participants and nonparticipants were compared (S1 Table). Baseline characteristics of women with PCOS who participated in the follow-up visit (*n* = 199) were similar to those who did not attend follow-up (*n* = 96), apart from higher age and lower total testosterone at baseline, whereas FG and other metabolic measurements were comparable.

### High rate of DM progression in PCOS

After a follow-up period of 10.6 ± 1.3 years, women with PCOS have become significantly more overweight, and the metabolic measurements in general have worsened, with increased use of medications (Table 1).

**Fig 1:**
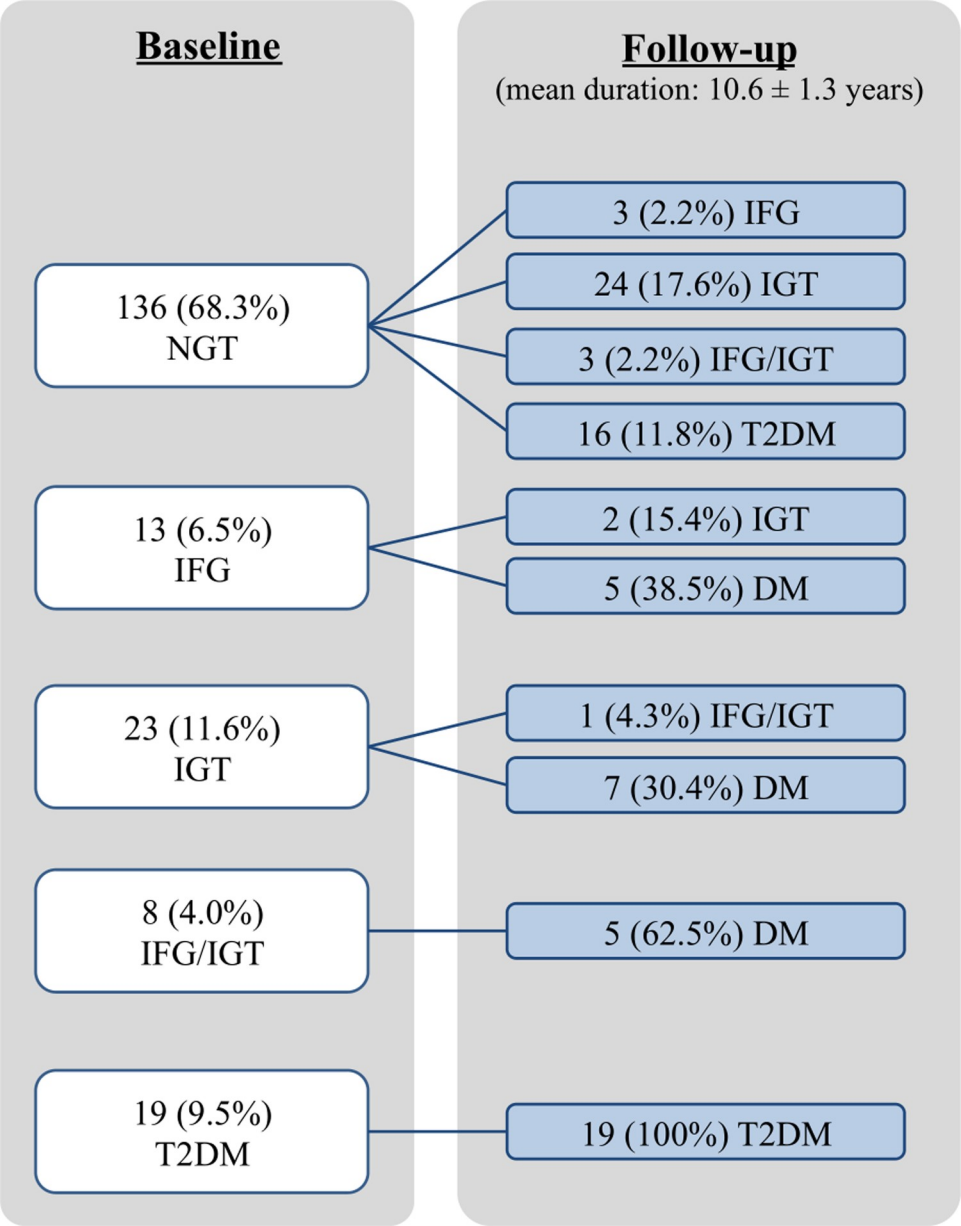
Progression of glycaemic status from baseline to follow-up with a mean duration of 10.6 ± 1.3 years in Chinese women with PCOS. Glycaemic status was defined according to the ADA 2009 guidelines. ADA, American Diabetes Association; IFG, impaired fasting glucose; IFG/IGT, combined impaired fasting glucose and impaired glucose tolerance; IGT, impaired glucose tolerance; NGT, normal glucose tolerance; T2DM, type 2 diabetes mellitus.

Over the follow-up period, the progression of impaired glucose regulation of women with PCOS was shown in Fig 1. Including both prevalent and incident cases, the frequency of impaired glucose regulation (IFG, IGT, IFG/IGT, or T2DM), T2DM, hypertension, and hyperlipidaemia of women with PCOS at follow-up has increased from 31.7% (95% CI 25.2%–38.1%) to 47.2% (95% CI 40.3%–54.2%), 9.5% (95% CI 5.5%–13.6%) to 26.1% (95% CI 20.0%–32.2%), 16.1% (95% CI 11.0%–21.2%) to 34.7% (95% CI 28.1%–41.3%), and 52.3% (95% CI 45.3%–59.2%) to 64.3% (95% CI 57.7%–71.0%), respectively. The mean age of T2DM onset was significantly lower in women with PCOS compared with women without PCOS (37.6 ± 6.2 versus 47.9 ± 8.4 years old, *p* < 0.001).

When we subcategorised women without PCOS into those with or without family history of T2DM, we found the cumulative incidence of T2DM at follow-up in women with PCOS was more or less similar to the data of women without PCOS but with a positive family history of diabetes (26.1% versus 25.0%, *p* > 0.05). Nevertheless, the cumulative incidence of T2DM at follow-up in women with PCOS was 2.5 times that of those without PCOS and without family history of T2DM (26.1% versus 10.6%, *p* < 0.05).

### Markedly higher incidence of T2DM among women with PCOS

A total of 19 out of 199 women with PCOS had T2DM at baseline (9.5%, 95% CI 5.5%–13.6%). Among women with PCOS free of T2DM at baseline, 36 out of 180 developed T2DM during follow-up (cumulative incidence of T2DM: 20% versus 9.8% in control) (S2 Table).

The crude and age-standardised incidence rates of T2DM among women with PCOS were 20.07 (95% CI 14.27–27.48) and 22.12 (95% CI 10.86–33.37) per 1,000 person-years, respectively (Table 2 and S1 Fig). Incidence rates of T2DM stratified by age at baseline increased steadily with age. Based on the available medical records, including information of women with PCOS who declined the follow-up visit but have a known diagnosis of T2DM at the time of follow-up would further increase the incidence rate to 24.37 per 1,000 person-years (95% CI 17.99–32.33).

**Table 2:** Comparison of incidence rate (95% CI) of type 2 diabetes mellitus per 1,000 person-years between Chinese women with PCOS, women without PCOS, and the Hong Kong general female population according to age and BMI category of participants.

	PCOS	Control	General female population^	Rate ratio (95% CI) between PCOS and control
**Sample size†**	**180**	**225**	**Approximately 3 million**	
**Crude incidence rate**	20.07 (14.27–27.48)	8.79 (5.65–13.10)	-	2.28 (1.34–3.88)**
**Age-standardised incidence rate§**	22.12 (10.86–33.37)	10.09 (4.92–15.26)	8.76 (8.72–8.80)	2.46 (2.44–2.48)***
**According to age groups (years):**				
10–19	13.13 (2.20–43.38)	-	-	-
20–29	19.50 (10.85–32.52)	6.62 (0.33–32.65)	0.58 (0.56–0.61)	2.95 (0.39–22.52)
30–39	20.56 (12.57–31.87)	6.81 (2.16–16.43)	1.88 (1.85–1.92)	3.02 (1.02–8.92)*
40–49	30.05 (7.64–81.79)	10.24 (5.70–17.07)	4.32 (4.27–4.38)	2.93 (0.84–10.30)
50–59	-	11.06 (3.51–26.67)	11.87 (11.77–11.97)	-
According to BMI categories:				
BMI < 23	8.96 (3.92–17.72)	4.86 (2.13–9.62)	-	1.84 (0.65–5.25)
BMI ≥ 23	28.64 (19.55–40.60)	14.1 (8.20–22.76)	-	2.03 (1.09–2.72)*

*p < 0.05.

**p < 0.01.

***p < 0.001.

Abbreviation: PCOS, polycystic ovary syndrome,

The age-standardised population incidence rate of T2DM among women in Hong Kong was 8.76 per 1,000 person-years (95% CI 8.72–8.80). Similarly, among 225 healthy women, the age-standardised incidence rate of T2DM was 10.09 per 1,000 person-years (95% CI 4.92–15.26). Among women aged 30–39 years, the age-stratified incidence rate of women with PCOS was 20.56 per 1,000 person-years (95% CI 12.57–31.87), which was approximately 10-fold higher than the age-stratified incidence rate of 1.88 per 1,000 person-years (95% CI 1.85–1.92) for women of that age range in the general Hong Kong population.

### Major determinants of the risk of progression to T2DM in PCOS

Based on WHO definition for obesity in Asians [17], women with PCOS were stratified into subgroups of normal weight (BMI < 23 kg/m^2^) or overweight/obese (BMI ≥ 23 kg/m^2^). At follow-up, overweight/obese women with PCOS had approximately 4-fold higher risk of T2DM compared with normal-weight women with PCOS (36.6% versus 9.2%, *p* < 0.05).

Incidence rates of T2DM stratified by BMI at baseline revealed overweight/obese women with PCOS had an approximately 3-fold higher risk of incident T2DM than normal-weight women with PCOS (28.64 [95% CI 19.55–40.60] versus 8.96 per 1,000 person-years [95% CI 3.92–17.72], *p* < 0.05) (Table 2). When compared with the corresponding BMI-matched women without PCOS, the incidence rate of T2DM of both normal-weight and overweight/obese women with PCOS was approximately double that of the respective control groups (normal weight: 8.96 [95% CI 3.92–17.72] versus 4.86 per 1,000 person-years [95% CI 2.13–9.62], *p* > 0.05; overweight/obese: 28.64 [95% CI 19.55–40.60] versus 14.1 per 1,000 person-years [95% CI 8.20–22.76], *p* < 0.05). The comparison between the normal-weight women with or without PCOS was not statistically significant, partly related to the small sample size with wide CIs and comparatively lower incidence rate in these subgroups with normal weight.

### Impact of different PCOS subphenotypes on progression to diabetes

Women with PCOS were categorized into phenotype A (all three Rotterdam criteria), phenotype B (hyperandrogenism and oligo- or anovulation), phenotype C (hyperandrogenism and polycystic ovarian morphology [PCOM]), and phenotype D (oligo- or anovulation and PCOM).

Incidence rate of T2DM based on the different PCOS subphenotypes was 24.88 (95% CI 14.61–35.15), 22.24 (95% CI 2.96–41.51), 0, and 13.77 per 1,000 person-years (95% CI 4.84–22.71), respectively (Table 3). Further stratification revealed incidence rate of T2DM among women with PCOS with hyperandrogenism was 1.7 times that of those without hyperandrogenism (23.67 [95% CI 14.85–32.50] versus 13.77 per 1,000 person-years [95% CI 4.84–22.71], *p* < 0.05).

**Table 3:** Comparison of incidence rate (95% CI) of type 2 diabetes mellitus per 1,000 person-years in Chinese women with PCOS according to different subphenotypes.

Phenotype	Total *N*	Period average (2003–2017), per 1,000 person-years	95% CI	
A	89	24.88	14.61	35.15
B	23	22.24	2.96	41.51
C	3	0	-	-
D	65	13.77	4.84	22.71

Phenotype A, all three Rotterdam criteria present; phenotype B, hyperandrogenism and oligo- or anovulation; phenotype C, hyperandrogenism and polycystic ovarian morphology; phenotype D, oligo- or anovulation and polycystic ovarian morphology.

Abbreviation: PCOS, polycystic ovary syndrome.

Multivariable logistic regression was performed combining data from Chinese women with PCOS and those of women without PCOS to examine the independent predictors for T2DM progression (Table 4). A diagnosis of PCOS was associated with 5.8-fold (95% CI 2.38–13.97, *p* < 0.001) higher risk of developing incident T2DM at follow-up, with adjustment for age at baseline and follow-up duration. In models 2 and 3, a diagnosis of PCOS remains an independent risk factor for incident T2DM after further adjustment for central obesity and elevated TG. However, in model 4, additional inclusion of FG rendered the association with PCOS nonsignificant.

**Table 4:** Logistic regression models for the risk of progression to diabetes in women with and without PCOS.

	Model 1		Model 2		Model 3		Model 4	
Variables	OR (95% CI)	*p*	OR (95% CI)	*p*	OR (95% CI)	*p*	OR (95% CI)	*p*
**Constant**	0.0002	<0.001	0.001	0.002	0.002	0.006	0.000002	<0.001
**Age**	1.05 (1.00–1.10)	0.04	1.03 (0.98–1.08)	0.274	1.03 (0.98–1.08)	0.328	1.00 (0.95–1.06)	0.934
**Follow-up duration**	1.46 (1.12–1.91)	0.01	1.35 (0.98–1.85)	0.067	1.30 (0.93–1.81)	0.119	1.11 (0.77–1.60)	0.594
**PCOS status**	5.77 (2.38–13.97)	<0.001	4.10 (1.62–10.35)	0.003	3.47 (1.34–8.95)	0.010	1.78 (0.63–5.03)	0.277
**WHR**			1.68 (1.25–2.25)	0.001	1.45 (1.06–2.00)	0.022	1.21 (0.84–1.75)	0.298
**Triglyceride§**					5.77 (1.52–31.95)	0.010	4.19 (0.98–17.96)	0.054
**Fasting glucose**							6.94 (3.44–14.00)	<0.001

Logistic regression model based on upon 180 observations from Chinese women with PCOS and 225 observations from women without PCOS: 19 out of 199 women with PCOS with T2DM and 17 out of 242 women without PCOS with T2DM at baseline were excluded. All variables that were not included in the model were not significant (even after transformation or categorisation when possible).

*WHR was expressed in its *z*-score, i.e., *z*-score = (WHR − mean)/SD.

§Triglyceride was log transformed because of a skewed distribution.

Abbreviations: OR, odds ratio; PCOS, polycystic ovary syndrome; T2DM, type 2 diabetes mellitus; WHR, waist-hip ratio.

Among women with PCOS, with adjustment for age at baseline and follow-up duration, a multivariable model identified that increased central obesity, reflected by *z*-score of WHR (OR = 1.71; 95% CI 1.08–2.69, *p* < 0.05), and elevated TGs (OR = 6.63; 95% CI 1.23–35.69, *p* < 0.05) are associated with progression to T2DM during follow-up (Table 5). We repeated the analysis in participants who have a full set of data (*n* = 168), and the results were essentially the same. Hyperandrogenism at baseline, with adjustment of age at baseline and follow-up duration, was associated with increased risk of T2DM progression. However, this association was rendered nonsignificant after further adjustment of either *z*-score WHR or BMI (S3 Table).

**Table 5:** Serial logistic regression models for risk of developing diabetes among Chinese women with PCOS.

		Model 1		Model 2		Model 3		Model 4		Model 5	
Variables	*N*	OR (95% CI)	*p*	OR (95% CI)	*p*	OR (95% CI)	*p*	OR (95% CI)	*p*	OR (95% CI)	*p*
**Constant**		0.08	0.007	0.001	0.001	0.009	0.039	0.00003	0.001	0.01	0.049
**Age**	180	1.04 (0.98–1.10)	0.201	1.05 (0.99–1.12)	0.088	1.02 (0.96–1.09)	0.526	1 (0.93–1.07)	0.980	1.03 (0.96–1.10)	0.438
**Follow-up duration**	180			1.41 (1.06–1.88)	0.018	1.26 (0.89–1.79)	0.195	1.05 (0.72–1.54)	0.793	1.23 (0.85–1.77)	0.276
WHR*	170					2.03 (1.32–3.11)	0.001	1.46 (0.91–2.33)	0.119	1.71 (1.08–2.69)	0.022
**Fasting glucose**	180							5.12 (1.99–13.15)	0.001		
**Triglyceride§**	168									6.63 (1.23–35.69)	0.028

Logistic regression model based on upon 180 observations: 19 out of 199 participants with type 2 diabetes at baseline were excluded. All variables that were not included in the model were not significant (even after transformation or categorisation when possible).

*WHR was expressed in its *z*-score, i.e., *z*-score = (WHR − mean)/SD.

§Triglyceride was log transformed because of a relevantly skewed distribution.

Abbreviations: OR, odds ratio; PCOS, polycystic ovary syndrome; WHR, waist-hip ratio.

## Discussion

We completed a long-term study with detailed metabolic characterisation of Chinese women with PCOS by using OGTT for comprehensive glycaemic status screening. Despite the limitations in study design, including the difference in age at baseline between women with and without PCOS, our long-term follow-up study has highlighted the high risk of progression to diabetes among women with PCOS. We found that the age-standardised incidence rate of T2DM among women with PCOS was around 2.5-fold higher compared with that of the control cohort and that of the local female population incidence rate. Diabetes onset was, on average, 10 years earlier among women with PCOS than in women without PCOS. Chinese women with PCOS were at 6-fold higher risk of developing T2DM compared with those without PCOS. High WHR, elevated TG, and presence of hyperandrogenism are associated with the progression to T2DM in PCOS.

There are only very few long-term prospective studies on the prevalence of T2DM in women with PCOS. A long-term prospective study on the prevalence of T2DM in an Italian PCOS population showed a rapid increase of 2.2% to 39.3% after a mean follow-up of 19 years [18]. Our study in Chinese women with PCOS noted a T2DM prevalence of 26.1% at the end of follow-up, which is comparable with the Italian study. This highlights the very significant lifetime risk of diabetes among women with PCOS. Nevertheless, another 10-year observational Australian study based on self-reported survey noted prevalence of T2DM increased from 2.0% to 5.1% in PCOS, which was much lower than that in the data shown in our current study [19]. The differences in T2DM prevalence among various studies might be due to the limitation of conducting a community/nationwide-based study, in which it would be difficult to perform OGTT in all participants [20], potentially leading to an underestimation of the diabetes risk among PCOS [21]. Moreover, studied populations and ethnicity of participants differed among the studies, which greatly affects reported phenotypic characteristics such as obesity [22–24] and thus contributes to the heterogeneity in the cardiometabolic risk in the different studies [20]. A recent systematic review regarding ethnicity, obesity, and the prevalence of IGT and T2DM has further highlighted that women with PCOS had an increased prevalence of IGT, with the Asian women having a 5.2-fold higher risk, the US women having a 4.4-fold higher risk, and European women having a 2.6-fold higher risk, and the prevalence was higher with obesity [8]. This suggested the risk for abnormal glucose tolerance might depend on the differential genetic underpinning [8], with Asian women expressing a higher metabolic risk [25].

Onset of T2DM at a young age imposes an early exposure to chronic hyperglycaemia and increases the susceptibility of progression to overt diabetes complications, including both microvascular and macrovascular [26]. Compared with the age- and sex-matched women without PCOS, patients with early onset of T2DM are associated with approximately 30-fold and 14-fold increased risk of developing cerebrovascular disease and myocardial infraction, respectively [27]. This evidence sheds some light on the potential burden of young-onset diabetes associated with PCOS.

In a recent review, the authors have summarised all the major longitudinal studies on cardiometabolic risk in women with PCOS searched from 1992 to 2018 on the PubMed electronic database [20]. Only investigations with follow‐up observation were taken into consideration. The authors took care to select observational research, excluding those that focused on the impact of medications or lifestyle interventions on clinical picture of PCOS. Moreover, all disorders relevant to pregnancy, delivery, and postpartum period in PCOS women, as well as reproductive and psychological problems and the occurrence of malignant diseases, were beyond the scope of this research and were not analysed. The review has summarised that a substantially higher incidence rate of developing T2DM in women with PCOS was noted in a long-term prospective study in Italy (PCOS versus general female population: 10.5 versus 0.4 per 1,000 person-years) [18]; a population, database-based study in the United Kingdom (PCOS versus women without PCOS: 5.7 versus 1.7 per 1,000 person-years) [28]; a retrospective cohort study in the UK (PCOS: 3.6 per 1,000 person-years) [29]; a population-based prospective cohort study in Iran (PCOS versus women without PCOS: 13.0 versus 4.0 per 1,000 person-years) [30]; and a national patient register-based study in Denmark (PCOS versus women without PCOS: 5.4 versus 1.6 per 1,000 person-years) [31] (S2 Fig). However, most of these studies did not include screening for T2DM in cases and women without PCOS using OGTT and thus would most likely have provided underestimates, especially among women without PCOS. Therefore, further research with the use of OGTT for comprehensive glycaemic status screening is indeed needed. In our current study, among women aged 30–39 years, the age-standardised incidence rate of T2DM among women with PCOS was approximately 3-fold compared to women without PCOS that were screened using OGTT but was 10-fold compared to the female population-based incidence rate (20.56 versus 6.81 versus 1.88). Of note, the population incidence rate was based on health records from the local public hospitals, often without OGTT screening. As a result, this figure would likely underestimate the true incidence rate, especially among the younger age groups. Ascertainment based on routine data capture from health records is highly dependent on people who attended public medical sectors and might have been influenced by case mix, patient demographics, and health-seeking behaviour [16]. In comparison, despite the relatively small sample size, our cohort of Chinese women with or without PCOS underwent screening for diabetes using standard 75-g OGTT during both the baseline and follow-up visit and therefore provided relatively reliable data on diabetes incidence. In agreement with a previous meta-analysis that highlighted that the OR for T2DM was 4.4 in women with PCOS compared with those without PCOS [4], we found that Chinese women with PCOS were at 5.8-fold risk of developing T2DM compared with women without PCOS in this study.

Previous studies have also revealed the association of obesity and the risk of abnormal glucose tolerance in women with PCOS. A recent prospective, population-based follow-up Finnish study suggested the high risk of T2DM in women with PCOS is mainly attributed to obesity, whereas normal-weight women with PCOS are not at increased risk for prediabetes or T2DM, and hence highlighted the synergistic effects of obesity and PCOS on T2DM [32]. This is in line with our BMI-stratified incidence data, whereby normal-weight women with PCOS have an incidence rate of T2DM similar to the overall incidence rate of our control cohort, whereas overweight/obese women with PCOS have a 3-fold higher incidence rate than normal-weight PCOS women have. In addition, our multivariable logistic regression model also suggested PCOS and central obesity were independently associated with progression to T2DM in our study. A recent Nordic cross-sectional study highlighted the prevalence of T2DM was not increased in normal-weight women with PCOS who have a BMI < 25 kg/m^2^ or waist < 88 cm [33]. This study, using a relatively young Nordic PCOS cohort (mean age: 29 years), suggested that routine OGTT screening may not be required in normal-weight PCOS women. However, our long-term prospective study with an older cohort of Chinese women with PCOS (mean age at follow-up: 41.2 years) found the cumulative incidence case of T2DM for lean PCOS has increased from 0% to 9.2% during the decade-long follow-up. In a recent international evidence-based guideline for the assessment and management of PCOS, published in the year 2018 [34], it is recommended that screening for T2DM should be considered in all adults with PCOS and in adolescents who are either overweight or from a high-risk ethnic group.

In our multivariable logistic regression model, an increase in *z*-score WHR and *z*-score BMI is associated with 1.9- and 1.8-fold risk of T2DM progression, respectively. This highlights that abdominal obesity, compared to general obesity, is of utmost importance in predicting the progression to T2DM [35], including in women with PCOS. This is in line with previous findings that suggested Asian women have a lower BMI in general compared with women of European descent, with abdominal obesity rather than BMI being the contributory role for the metabolic disturbances seen among Asian women [36]. Because of obesity and IR, hyperinsulinemia in women with PCOS would further elevate the bioavailable androgen levels through lowering SHBG level [37]. The significantly elevated bioavailable androgen levels are thought to contribute to the metabolic abnormalities in women with PCOS [38]. Yet, to date, it is still debatable whether women with PCOS with hyperandrogenism have worse clinical and metabolic profile than those without [39]. Our data suggested hyperandrogenism was associated with approximately 3-fold risk of incident T2DM. However, the effect of hyperandrogenism on the risk of incident T2DM was rendered nonsignificant after adjusting for abdominal obesity, suggesting that some of the adverse metabolic effects of hyperandrogenism in PCOS may be mediated by central obesity [37,40]. However, since we are unable to study the effect of IR on the progression of diabetes in women with PCOS in this model, we could not address whether the impact of androgens is independent of IR or not. Therefore, further research is clearly warranted.

Our study has several limitations. Our sample size was moderate, with a limited number of incident diabetes during the follow-up period. Women with PCOS were recruited from the medical, endocrine, and gynaecology clinics of a tertiary referral centre. This may introduce some potential selection bias with overrepresentation of women with PCOS with medical problems or at risk of metabolic disorders. In this study, baseline data of fasting insulin were not available in a number of participants, which hindered the possibility of exploring the effect of fasting insulin and HOMA-IR in the regression models. Immunoassay was used to measure total testosterone of women with PCOS at baseline, whereas LC-MS/MS was used at the follow-up assessment. Notably, experts have suggested radioimmunoassay (RIA) for total testosterone is comparable to LC-MS assays; yet significant variability is present at low testosterone levels between LC-MS assays and poor precision with all assays [41]. In addition to the fact that total testosterone levels will be lowered as women with PCOS age, using a higher-sensitivity and higher-specificity LC-MS/MS assay at our follow-up assessment might further lower the total testosterone level measured compared with the baseline data. Therefore, a conversion calculation was performed in order to provide comparisons between the baseline and follow-up total testosterone results. Hirsutism is a key clinical sign for hyperandrogenism [42]. However, a significant proportion of our women with PCOS have undergone semipermanent hair-removal laser treatment, which hindered the Ferriman-Gallwey scoring [42]. Although FAI was measured in this study and is being considered, it was not used to define hyperandrogenism, because there was no universal guideline regarding FAI and because a standard cutoff for defining hirsutism according to FAI is not readily available. The current international guidelines note that FAI cutoffs from reference laboratories are recommended as the cutoff values. Therefore, the failure to include FAI as a measure of hyperandrogenism is indeed a limitation in this study. Of note, our control cohort was significantly older than the cohort of women with PCOS. Nevertheless, a sensitivity analysis restricting cases and controls to the overlapping age range of 20–43 years yielded similar findings (S4 Table). Moreover, we believe that the participants included as controls are an appropriate comparison group because they were recruited from a population-based study of healthy women and have undergone the same assessment at similar times at both baseline and follow-up as the PCOS cohort. Furthermore, given the significant differences in age at recruitment for participants with PCOS versus controls, this difference in age at baseline might have contributed to the observed differences during follow-up in the proportion with young-onset diabetes noted in women with PCOS. Based on the available electronic medical records, some of the women with PCOS have progressed to T2DM during the follow-up period yet have declined to return for a complete follow-up assessment at the research centre. Therefore, the frequency and incidence rate of T2DM have been underestimated. The progression of prediabetes and T2DM is also highly dependent on ethnicity [24]; thus, the findings in our study might not be applicable to other populations.

To conclude, we investigated the progression of glycaemic status among Chinese women with PCOS (mean age: 41.2 ± 6.4 years) and compared those data with those from women without PCOS (mean age: 54.1 ± 6.7 years), by using OGTT for comprehensive evaluation of glycaemic status. Despite the limitations (including a modest sample size and potential selection bias of women with PCOS), to the best of our knowledge, this is one of the first long-term follow-up studies in Asia that highlighted the high age-standardised incidence rate of T2DM among PCOS women (22.12 per 1,000 person-years; 95% CI 10.86–33.37), which was further compared with the incidence rate in the local female population (8.76 per 1,000 person-years; 95% CI 8.72–8.80). We have also highlighted that Chinese women with PCOS were at 6-fold increased risk of developing T2DM compared with those without PCOS. Our data suggested WHR, elevated TG, and hyperandrogenism are associated with the progression to T2DM in PCOS, although we were unable to explore relationships with IR. Given the progression of cardiometabolic risk across the life span of Chinese women with PCOS, we highlighted their long-term healthcare risks. Prior abstract presentation of our data had been considered and incorporated into the recent international guidelines, suggesting the clinical relevance of this work. We believe our data provide useful guidance towards formulating metabolic screening recommendations, as well as highlighting the need for development of a multidisciplinary approach to the management of these high-risk participants.

## Supporting information

S1 Strobe checklist STROBE checklist for the study. Strobe, Strengthening the Reporting of Observational Studies in Epidemiology.
(DOC)

S1 Doc Research protocol for the study. (DOCX)

S2 Doc Questionnaire for the study. (DOCX)

S1 Table Baseline characteristics of Chinese women with PCOS who participated in follow-up study and those that defaulted. PCOS, polycystic ovary syndrome.
(XLSX)

S2 Table Comparison of baseline characteristics of PCOS DM converters versus PCOS non-DM converters, at baseline (t0) and at follow-up (t1)*. DM, diabetes mellitus; PCOS, polycystic ovary syndrome.
(XLSX)

S3 Table Serial logistic regression models for risk of diabetes among Chinese women with PCOS. PCOS, polycystic ovary syndrome.
(XLSX)

S4 Table Logistic regression models for the risk of progression to diabetes in women with and without PCOS, including participants aged 20–43 years only. PCOS, polycystic ovary syndrome.
(XLSX)

S1 Fig Crude and age-standardised incidence rate (95% CI) of T2DM per 1,000 person-years between Chinese women with PCOS (*n* = 180, baseline mean age = 30.2 ± 6.5 years), women without PCOS (*n* = 225, baseline mean age = 42.6 ± 7.1 years), and the Hong Kong general female population (*n* = approximately 3 million), as well as the incidence rate according to age category of participants were shown. **Glycaemic status was captured through 75-g OGTT in Chinese women with PCOS and women without PCOS, whereas that was ascertained through public sector electronic health records in the Hong Kong general female population.** OGTT, oral glucose tolerance test; PCOS, polycystic ovary syndrome; T2DM, type 2 diabetes mellitus.
(TIF)

S2 Fig Comparison of crude incidence rate (95% CI) of T2DM per 1,000 person-years in different accessible studies. T2DM, type 2 diabetes mellitus.
(TIF)

## Abbreviations

AMH: anti-Müllerian hormone
BP: blood pressure
DM: diabetes mellitus
FAI: free androgen index
FG: fasting glucose
FSH: follicle-stimulating hormone
HDL-C: high-density lipoprotein cholesterol
HOMA-IR: homeostatic model assessment of insulin resistance
IFG: impaired fasting glucose
IGR: impaired glucose regulation
IGT: impaired glucose tolerance
IR: insulin resistance
LC-MS/MS: liquid chromatography tandem mass spectrometry
LDL-C: low-density lipoprotein cholesterol
LH: luteinizing hormone
MetS: metabolic syndrome
NGT: normal glucose tolerance
OGTT: oral glucose tolerance test
OR: odds ratio
PCOM: polycystic ovarian morphology
PCOS: polycystic ovary syndrome
PWH: Prince of Wales Hospital
RIA: radioimmunoassay
SHBG: sex hormone–binding globulin
T2DM: type 2 diabetes mellitus
TC: total cholesterol
TG: triglyceride
WHR: waist-to-hip ratio
