# Supplementary material for: Progression of glucose intolerance and cardiometabolic risk factors over a decade in Chinese women with polycystic ovary syndrome: A case-control study
Source: PLoS Med. 2019 Oct 25;16(10):e1002953. doi: 10.1371/journal.pmed.1002953 (PMC6814217; doi:10.1371/journal.pmed.1002953)
Supplement: S1 Doc — (DOCX) [file pmed.1002953.s002.docx]

Research Proposal

**Progression of glucose intolerance, cardiometabolic risk factors and subclinical atherosclerosis in Chinese women with Polycystic Ovary Syndrome**

Principal Investigator: Ma, Ching Wan Ronald

Co-investigators: Cheung, Lai Ping

Li, Tin Chiu

Huang, Jin

Liu, Kin Hung

Abstract

The prevalence of type 2 diabetes has increased markedly over recent decades, and diabetes has become a global public health problem. An increasing proportion of young adults are diagnosed with diabetes, putting them at risk of long-term diabetic complications and increase in healthcare burden. Polycystic ovary syndrome is the commonest endocrine disorder affecting women of reproductive age, and in addition to menstrual irregularity and fertility problems, is associated with increased insulin resistance and marked increase in the risk of type 2 diabetes. In addition to impaired glucose tolerance and diabetes, subjects with PCOS also have increased risk of hypertension, hyperlipidaemia, as well as other cardiometabolic risk factors, when compared to subjects without PCOS. There is much recent debate regarding the cardiovascular risk in women with PCOS, especially in relation to the risk profile as the patients go beyond the reproductive age. It is also not clear which subphenotype of PCOS is most at risk of cardiometabolic problems. Our group has established a registry of over 300 Chinese patients with PCOS, with detailed documentation of baseline characteristics and metabolic evaluation. We aim to conduct a comprehensive follow-up study of these PCOS subjects, in order to address some of the current knowledge gaps. In this study, we aim to evaluate the rate of progression to glucose intolerance/type 2 diabetes, as well as changes in cardiometabolic risk factors, approximately 10 years after the baseline evaluation. We will also assess for evidence of early atherosclerosis by measuring carotid intima media thickness (IMT). The changes of reproductive profile of PCOS women will also be assessed, such as menstruation, hormone and ultrasound parameters of ovary and uterus. All results will be compared to a control group of subjects who have undergone the same metabolic and imaging evaluation at baseline, with recent follow-up evaluation completed approximately 10 years after the baseline assessment. In addition to comparing the incident rate of glucose intolerance/diabetes, we also aim to compare the metabolic risk and the changes in reproductive profile of the different PCOS subphenotype. Finally, we will evaluate the ability of visceral adiposity and adipokines to predict the risk of incident diabetes among women with PCOS. Results from our proposed study will help inform the planning of metabolic screening and monitoring of cardiovascular risk factors among Chinese women with PCOS, and to facilitate earlier detection and optimal management of this high-risk population to reduce the risk of progression to diabetes and its associated complications.

Objectives

1. To evaluate the progression of dysglyaemia in Chinese women with PCOS
2. To evaluate the development of other cardiometabolic risk factors in women with PCOS
3. To compare changes in carotid intima-media thickness as a marker of progression of subclinical atherosclerosis in PCOS and control subjects
4. To compare the risk of metabolic abnormalities among women with different sub-phenotype of PCOS
5. To evaluate the role of visceral adiposity and adipokines in predicting incident diabetes and glucose intolerance in PCOS
6. To compare the changes in reproductive profile of PCOS women with control subjects

2a. Background of research:

(A) Work done by others

**2.1 Epidemiology of Diabetes in Asia**

There is a global epidemic of diabetes, with the situation particularly alarming in Asia. In China, the prevalence of diabetes has increased dramatically from around 1% in 1980 to the latest figure of 11.6% from a nationwide survey [^1^](#_ENREF_1) [^2^](#_ENREF_2). In a systematic review of 22 studies on diabetes prevalence and associated factors in China from 2000-2010, diabetes prevalence increased from 2.6% to 9.7% during this decade, with increasing age, urban residence, positive family history, obesity and hypertension being common associated risk factors for type 2 diabetes [^3^](#_ENREF_3). Of particular concern is the increasing proportion of young adults affected. In a recent nationwide epidemiology study from China, the proportion of women aged 30-39 affected with diabetes was 3%, with another 9.2% of women in that age group affected by impaired glucose tolerance [^4^](#_ENREF_4).

**2.2 Polycystic Ovary Syndrome as an important cause for glucose intolerance and young-onset type 2 diabetes**

Type 2 diabetes results from complex interactions between multiple genetic susceptibility factors, as well as environmental and behavioural factors. Both insulin resistance and beta-cell dysfunction are important in the pathogenesis of type 2 diabetes. Polcystic Ovary Syndrome, a common endocrine disorder affecting women of reproductive age, has recently emerged as an important risk factor for type 2 diabetes [^5^](#_ENREF_5) [^6^](#_ENREF_6). PCOS is also associated with a significantly increased risk for gestational diabetes [^6^](#_ENREF_6) [^7^](#_ENREF_7).

Polycystic Ovary Syndrome (PCOS) is a common condition affecting women of reproductive age, with reported prevalence of 6%-21%, depending on the diagnostic criteria applied and population being studied [^8^](#_ENREF_8). In a recent large-scale epidemiology study in China, prevalence of PCOS was found to be 5.6% [^9^](#_ENREF_9). PCOS is characterized by chronic anovulation and manfestations of excess androgens [^10^](#_ENREF_10)^,^[^11^](#_ENREF_11). In addition to the well-known gynaecological complications, it is well-established that women with PCOS are also at increased risk of a metabolic disoders, and have 2-5 fold increased risk of diabetes [^11^](#_ENREF_11). Both obese and lean women with PCOS have increased insulin resistance and impaired β-cell function when compared to age- and body mass index-matched controls [^12^](#_ENREF_12). Even among lean patients with PCOS, 31% have impaired glucose tolerance (IGT) and 7.5% have type 2 diabetes [^13^](#_ENREF_13). The conversion from IGT to DM is also accelerated in PCOS [^14^](#_ENREF_14)^,^[^15^](#_ENREF_15). In a long-term prospective study from Italy, in which 225 women with PCOS were followed for mean duration of 16.9 years, the incidence rate of type 2 diabetes in the study population was 1.05 per 100 person-years, compared to 0.4 per 100 person-years in the general population [^16^](#_ENREF_16). The age-standardized prevalence of diabetes at the end of follow-up was alarmingly at 39.3%, compared to a rate of 5.8% in the general population of a similar age.

**2.3 Increased cardiovascular risk in young women with PCOS**

In addition to increased risk of diabetes, women with PCOS have increased risk of hypertension, hyperlipidaemia, central obesity and obstructive sleep apnoea, as well as metabolic syndrome [^17^](#_ENREF_17) [^11^](#_ENREF_11)^,^[^18^](#_ENREF_18). In the US, approximately 50% of women with PCOS fulfil the diagnostic criteria for the metabolic syndrome (MES) [^11^](#_ENREF_11). This clustering of cardio-metabolic risk factors in young women with PCOS suggest they are at increased cardiovascular risk. Early studies which compared carotid intima-media thickness in women with PCOS compared with controls suggested that women aged >45 with PCOS have significantly higher carotid IMT compared with controls, highlighting increased risk for subclinical atherosclerosis after prolonged exposure to an adverse cardiovascular risk profile [^19^](#_ENREF_19).

**2.4 Subphenotype of PCOS and the different clinical features**

Women with hyperandrogenic PCOS appear to have a worse cardiometabolic profile and higher prevalence of cardiovascular risk factors compared with women with non-hyperandrogenic PCOS [^20^](#_ENREF_20) [^21^](#_ENREF_21) [^22^](#_ENREF_22) [^23^](#_ENREF_23) [^24^](#_ENREF_24). Given the accumulating evidence of the importance of hyperandrogenism, there has been increasing emphasis to consider PCOS as a disorder of hyperandrogenaemia, and to identify the hyperandrogenic PCOS phenotype as a separate entity [^25^](#_ENREF_25).

Despite these controversies, at the most recent NIH Evidence-based methodology Workshop on PCOS in Dcember 2012, the recommendation from an independent panel of experts was to maintain the broad diagnostic criteria established by the Rotterdam criteria, but also to encourage the use of distinct phenotyping in future research, in order to characterize the cardiometabolic implications of the individual PCOS phenotypes [^26^](#_ENREF_26). This emphasis on the need to clarify the metabolic risk of different subphenotypes of PCOS is in line with the conclusion of the European Society for Human Reproduction and Embryology and American Society for Reproductive Medicine-endorsed third PCOS consensus meeting in Amsterdam [^27^](#_ENREF_27), highlighting this gap in our current knowledge that requires further research.

In a recent cross-sectional multi-centre study including 2,288 well phenotyped women with PCOS from the Netherlands, it was noted that women with hyperandrogenic PCOS had a worse cardiometabolic profile and a higher prevalence of cardiovascular risk factors including obesity and insulin resistance when caompared to women with nonhyperandrogenic PCOS. Nevertheless, obesity and dyslipidaemia was highly prevalent even in non-hyperandrogenic PCOS, highlighting the need for regular screening of cardiometabolic risk factors among all women with PCOS [^24^](#_ENREF_24).

**2.5 Natural history of PCOS**

In the normal population, both abdominal obesity and insulin resistance tend to increase with advancing age, and thus the risk of cardiovascular disease and metabolic syndrome increase with aging. In PCOS, abdominal obesity and the prevalence of metabolic syndrome increase with increasing age [^28^](#_ENREF_28) [^29^](#_ENREF_29). Indeed, in a recent survey of 357 members of the European Society of Endocrinology, it was noted that among endocrinologists, obesity and type 2 diabetes remain the principal long-term concerns for physicians caring for patients with PCOS [^30^](#_ENREF_30).

Whilst it is well known that PCOS is associated with increased risk of diabetes, recent studies suggest that cardiovascular risk in PCOS women may be normalized with increasing age [^31^](#_ENREF_31). In a study of older postmenopausal women who were affected by PCOS during their reproductive years, it was noted that the prevalence of type 2 diabetes normalized with aging (age 60-70) in PCOS [^32^](#_ENREF_32). Likewise, it has been suggested that the cardiovascular risk of PCOS may decrease with increasing age, partly due to the decline in ovarian androgen production with age [^33^](#_ENREF_33)^,^[^34^](#_ENREF_34). Another possible contributing factor to normalization of risk is occurrence of ovulatory cycles, which was noted to occur with increasing age in around 30% of women with PCOS in Italy [^33^](#_ENREF_33). The natural history of PCOS in Chinese is largely unknown. Given this paucity of data, our proposed research will help provide much-needed information on the natural history of PCOS in Chinese, and help inform future planning of healthcare services for this group of women.

(B) Work done by our research group

**2.6 Healthcare implications of young-onset diabetes**

Our group has made significant contributions to the study of diabetes epidemiology among Asians [^35^](#_ENREF_35) [^36^](#_ENREF_36) [^37^](#_ENREF_37) [^38^](#_ENREF_38), and have highlighted the potential impact of increasing number of patients with young onset diabetes [^39^](#_ENREF_39)^,^[^40^](#_ENREF_40). In the Hong Kong Diabetes Registry, overweight subjects with type 2 diabetes diagnosed before the age of 40 was found to have significantly higher risk of developing cardiovascular and renal complications compared with normal weight subjects with early-onset type 2 diabetes, or subjects with type 1 diabetes, highlighting the high risk of complications and potential healthcare burden of this group [^39^](#_ENREF_39). In the Joint Asia Diabetes Evaluation (JADE) Programme, among 41029 subjects with type 2 diabetes from 9 Asian countries/regions, around 20% of subjects have diabetes before age of 40 [^41^](#_ENREF_41). Increasing number of women with onset of diabetes during the reproductive period will also impact on the number of pregnancies complicated by hyperglycaemia, and together with increasing prevalence of GDM, further contribute to the epidemic of diabetes and obesity through its adverse metabolic impact on the offspring [^42^](#_ENREF_42)^,^[^43^](#_ENREF_43).

**2.7 Metabolic risk in Chinese women with PCOS**

The long-term healthcare needs of Chinese women with PCOS is largely unknown. In one of the first major study from Asia examining cardio-metabolic risk factors among subjects with PCOS, we evaluated 295 premenopausal Chinese women with PCOS diagnosed by the Rotterdam criteria (mean age: 30.2±6.4 years) and 98 control subjects without PCOS for presence of MES and cardiovascular risk factors. Alarmingly, 21.4% of the PCOS subjects had dysglycaemia (i.e. impaired fasting glucose, impaired glucose tolerance or type 2 diabetes), compared to 7.1% in the control subjects. Metabolic syndrome according to the updated ATPIII 2005 criteria [^44^](#_ENREF_44) was found in 24.9% of PCOS women, compared to 3.1% of controls. The frequency of MES increased with increasing age of the subjects, but was consistently higher among subjects with PCOS compared to controls. The prevalence of MES in PCOS women increased from 16.7% at under 30 years of age to 53.3% at over 40 years. The frequency of each MES component, in decreasing order, was central obesity (53.1%), elevated blood pressure (29.4%), reduced HDL-C (28.6%), increased TG (21.4%) and impaired fasting glucose (21.4%). Increasing age, higher BMI and a diagnosis of PCOS were found to be independent predictors of metabolic syndrome in multivariate logistic regression analysis, with PCOS associated with a 5-fold increase in the risk of MES (OR 4.90; 95% CI: 1.35-17.84) even after adjustment for age and BMI [^28^](#_ENREF_28). Furthermore, we noted that young PCOS subjects with metabolic syndrome have increased carotid intima-media thickness compared with PCOS subjects without cardiometabolic risk factors, suggesting early atherosclerotic disease [^45^](#_ENREF_45).

Our group has subsequently examined the role of hyperandrogenism and metabolic risk in Chinese women with PCOS. In an earlier analysis of 197 PCOS subjects from the PWH PCOS Registry diagnosed according to the Rotterdam criteria, only 63% (125 of 195) were anovulatory hyperandrogenic, and thus fulfilled the NIH criteria. When these PCOS subjects with hyperandrogenism were compared to the remaining 72 anovulatory normoandrogenic women with polycystic ovaries or ovulatory hyperandrogenic women with polycystic ovaries, it was noted that PCOS subjects who fulfill the NIH criteria, despite being younger, were more likely to have central obesity, and were more insulin resistant, suggesting that PCOS subjects with both hyperandrogenism and anovulation are most at risk of metabolic abnormalities [^23^](#_ENREF_23).

In the recent large epidemiological study in China, among the 833 women diagnosed to have PCOS, metabolic syndrome was present in 26.8% [^9^](#_ENREF_9). The alarming prevalence of cardiometabolic risk factors among young women with PCOS suggests this is a problem with significant public health impact. Our proposed project will provide much needed information on the progression of these cardio-metabolic risk factors among young women with PCOS, and will form the basis for formulating screening recommendations as well as development of a multidisciplinary approach to the management of these high-risk young subjects.

**2.8 Role of visceral adiposity in the pathogenesis of metabolic disorders in PCOS and sonographic measurement of mesenteric fat thickness**

Visceral adiposity is recognized as a major risk factor for diabetes, and tendency to central and visceral adiposity may partly explain the increased risk of diabetes observed among Asians [^38^](#_ENREF_38). Nevertheless, the need for expensive and time-consuming imaging modalities such as computer tomography and magnetic resonance imaging (MRI) has limited the ability to evaluate visceral adiposity in large epidemiological studies. Our group (Dr Eric Liu) has pioneered the use of sonographic measurement of mesenteric fat as a non-invasive tool to evaluate visceral adiposity accurately [^46-49^](#_ENREF_46). When compared to MRI measurement of total abdominal and visceral fat, sonographic measurement of mesenteric fat thickness showed better associations with some of the cardiovascular risk factors, highlighting its utility to evaluate regional fat distribution in the assessment of cardiovascular risk [^46^](#_ENREF_46). In our previous study of cardiovascular risk in Chinese PCOS subjects, we found that mesenteric fat thickness as a measure of visceral adiposity was strongly correlated to glucose intolerance, cardiometabolic risk and carotid intima-media thickness [^45^](#_ENREF_45), and was an independent predictor for the presence of non-alcoholic fatty liver disease (NAFLD) [^50^](#_ENREF_50). In our subsequent cross-sectional comparison of visceral adiposity and metabolic risk in PCOS and non-PCOS controls, among 117 PCOS subjects and 415 non-PCOS controls who underwent the US measurements, we noted that PCOS women had significantly higher mesenteric fat thickness. Furthermore, on multivariate linear regression using carotid intima-media thickness as the outcome variable, independent variables predicting carotid IMT included age, a diagnosis of PCOS, and mesenteric fat thickness, highlighting the potential relationship between visceral adiposity and cardiovascular risk among women with PCOS [^51^](#_ENREF_51).

Several adipokines including adiponectin, visfatin and omentin-1 have been found to be associated with visceral adiposity and cardiovascular risk. We have previously found good correlation between adiponectin, omentin-1 and visceral adiposity as measured sonographically using mesenteric fat thickness [^52^](#_ENREF_52) [^53^](#_ENREF_53). We have also noted increased levels of visfatin among PCOS subjects [^54^](#_ENREF_54). In this project, we will also compare the discriminatory ability of baseline measurements of visceral adiposity, other clinical parameters, as well as baseline adipokine levels in the prediction of incident diabetes on follow-up.

**2.9 Menstruation, hormone and ultrasound parameters of ovary and uterus**

Patients with PCOS usually present with menstrual disturbances, infertility, increased hair growth and acne. Their ovaries usually have characteristic ultrasound features (e.g. increased ovarian volume and blood flow). Our previous findings of more antral follicles, larger total ovarian volume and stromal volume, and increased ovarian vascularity were consistent with the typical ultrasound feature. Our observations of increased stromal vascularity and volume or echogenicity in hirsute or non-obese PCOS women respectively was compatible with the pathogenic role of ovarian stroma and had highlighted the importance of objective stromal quantification by ultrasonography. However, the changes of features with age have not been well delineated. The ultrasound appearance of ovary and uterus will also be compared among different PCOS sub-phenotype and with the controls.

2b) Research plan and methodology

**Subjects**

We plan to conduct a detailed clinical and metabolic evaluation of a cohort of women previously diagnosed to have PCOS at the Combined Gynaecology-enodcrinology clinic at the Prince of Wales Hospital (PWH), and enrolled into our PWH PCOS Registry. This cohort includes around 300 Chinese pre-menopausal women with PCOS were previously recruited between July 2003 and April 2007 into a cross-sectional study of prevalence of cardiometabolic risk factors in PCOS, as well as additional cases recruited since 2007. All subjects fulfilled a diagnosis of PCOS based on the 2003 Rotterdam consensus [^55^](#_ENREF_55) with at least two of the following features: (i) oligo-amenorrhoea or chronic anovulation, (ii) clinical and/or biochemical hyperandrogenism, (iii) ultrasound appearance of polycystic ovaries, after exclusion of other known causes of hyperandrogenaemia. Exclusion criteria will include the use of medications which are known to affect steroid or glucose metabolism (e.g. oral contraceptives, corticosteroids) within three months before baseline visit. Subjects with hypothyroidism, prolactinoma, non-classical adrenal hyperplasia and Cushing’s syndrome will also be excluded from the study.

Control subjects will be from a group of healthy women recruited from a community-based study of cardiometabolic risk [^56^](#_ENREF_56). Control subjects have no clinical or biochemical evidence of hyperandrogenism, and have no history of oligomenorrhoea or chronic anovulation. Control subjects have undergone the same detailed metabolic evaluation as subjects with PCOS, including OGTT at baseline. The majority of the subjects have also had sonographic measurement of mesenteric fat thickness and carotid IMT at baseline [^49^](#_ENREF_49). The control group is currently being evaluated as part of another project evaluating the rate of progression of diabetes and cardiometabolic risk in community-recruited healthy subjects. All control subjects will undergo the same metabolic evaluation as the PCOS subjects, including a 75g OGTT. Mesenteric fat measurement and carotid IMT will also be re-measured by the same operator on follow-up. Among 65 female control subjects (age at baseline: 34.0±5.2 [range 20-40], BMI 22.7±3.6kg/m^2^) with normal blood glucose who have completed the follow-up assessment, only 3 people (4.6%) developed diabetes after a median follow up period of 11.5 (IQR: 11.1-11.8) years. The evaluation of the control subjects is ongoing, and given the similar duration of follow-up from the baseline evaluation, will provide a control population for comparison of rate of progression of glucose intolerance and cardiometabolic risk. The current study protocol will conform to the Declaration of Helsinki, and will be approved by the Clinical Research Ethics Committee of the Prince of Wales Hospital, Shatin. Written informed consent will be obtained from each participant invited to take part in the study.

**Methods and Study Design**

Subjects in the PCOS Registry and control subjects will be invited to take part in the follow-up study. A standard questionnaire will be used to document personal, medical and drug history, regularity and length of menstrual cycles, ovulation status, symptoms of hirsutism and acne. Cardiovascular risk factors such as tobacco and alcohol use, physical inactivity and family history of diabetes will also be recorded. Physical inactivity will be defined as moderate physical activity of less than 30 minutes per week. Reproductive history including history of previous pregnancies and use of fertility treatment will also be recorded. Signs of androgen excess will be noted in the physical examination and graded according to the Ferriman-Gallwey score. Body weight (kg), body height (m), waist and hip circumferences (cm) will be measured. Waist circumference will be taken as the narrowest measurement midway between the top of the iliac crest and the lower rib margin while the hip circumference was taken as the widest measurement at the level of the greater trochanters. Sitting blood pressure will be measured after a 5-minute rest using a standard sphygmomanometer. Overnight fasting blood specimens will be obtained in all women for measurements of fasting plasma glucose (FPG), total cholesterol (TC), triglycerides (TG) and high-density lipoprotein cholesterol (HDL-C), as well as renal function, liver function and urate. Serum total testosterone, androstenedione and sex-hormone binding globulin will be measured. Fasting insulin will be measured using Immulite (Siemens Diagnostics Inc.). In addition, a standard 75g-oral glucose tolerance test will be performed in all subjects to evaluate for development of glucose intolerance.

Subjects will be classified according to presence and absence of dysglycaemia and abnormal cardiometabolic risk factors. Other outcome variables include development of dyslipidaemia and hypertension. Dysglycaemia will be defined according to the World Health Organization (WHO) 2006 definition using 75g OGTT. These include impaired fasting glucose (IFG: FPG ≥ 6.1 and <7 mmol/L), impaired glucose tolerance (IGT: 2-hour PG ≥ 7.8 and <11.1 mmol/L), and type 2 diabetes (FPG ≥ 7 mmol/l or 2-hour PG ≥ 11.1 mmol/L or treatment with anti-diabetic medications). Hypertension is defined based on the BP treatment thresholds proposed in the recent report from the Panel Members appointed to the Eighth Joint National Committee (JNC8) as SBP ≥ 140 mmHg or DBP ≥ 90mm Hg [^57^](#_ENREF_57). Metabolic syndrome (MES) will be defined according to the modified National Cholesterol Education Program Adult Treatment Panel III criteria proposed by the American Heart Association and the National Heart, Lung and Blood Institute (AHA/NHLBI) in 2005 (ATPIII2005). Metabolic syndrome is defined as the presence of 3 or more of the following risk factors: i) Central obesity defined using ethnic-specific cut-offs for Chinese, with waist circumference ≥ 80cm; ii) elevated systolic and/or diastolic blood pressure ≥ 130/85mmHg or the use of anti-hypertensive medications; iii) elevated fasting triglycerides ≥ 1.7mmol/l or the use of lipid-lowering agents; iv) reduced high-density lipoprotein cholesterol (HDL) <1.3mmol/l or the use of lipid-lowering treatment; v) impaired fasting glucose ≥ 5.6mmol/l or the use of glucose-lowering treatment. In addition to MES, dyslipidaemia is defined by TC ≥ 5.2 mmol/L, TG ≥ 1.7 mmol/L, HDL-C <1.3 mmol/L, LDL-C ≥ 3.4 mmol/L, or treatment with lipid regulating drugs.

Follow-up sonographic measurements of mesenteric fat thickness, carotid IMT, aortic stiffness, renal resistance index and sonographic assessment of ovaries and uterus will be conducted using the same equipment and protocol as to the baseline measurements [^49^](#_ENREF_49)^,^[^50^](#_ENREF_50). A selected subgroup will undergo 3-D ultra-sound to evaluate ovarian morphology. A follow-up of menstruation changes and reproductive history will be assessed by questionnaires. The study is in compliance with the Declaration of Helsinki and ICH-GCP.

**Analysis plan:**

To address **Objective 1**, the rate of progression to abnormal glucose tolerance and diabetes will be calculated for the subgroup of PCOS subjects with normal glucose tolerance at baseline. Both the cumulative incidence as well as the incidence rate (expressed as the number of cases per 100 person-years) will be computed and compared with the incidence and incidence rate of abnormal glucose tolerance among non-PCOS control subjects.

For **Objective 2** and **Objective 3**, changes in cardiometabolic risk factors and carotid intima-media thickness will be compared between PCOS cases and controls.

In order to examine the impact of different sub- phenotypes of PCOS on metabolic risk as outlined in **Objective 4**, PCOS subjects in the Registry will be further divided according to the following sub-phenotypes suggested by the NIH:

1. androgen excess + ovulatory dysfunction (AE+ OD)

2. androgen excess + polcystic ovarian morphology (AE + PCOM)

3. Ovulatory dysfunction + polycystic ovarian morphology (OD+ PCOM)

4. Androgen excess + ovulatory dysfunction + polycystic ovarian morphology (AE+ OD+ PCOM)

Women belonging to sub-phenotype 1,2 and 4 will be classified as hyperandrogenic(HA), while women belonging to phenotype 3 will be defined as non-HA PCOS, and comparison of cardiometabolic risk factors between the two groups will be made.

To address **Objective 5**, baseline measurements of mesenteric fat thickness, as well as measurements of adipokines including adiponectin and omentin-1, will be entered into a multivariate model to examine their independent ability to predict development of glucose intolerance. In addition, we will utilize ROC analysis to examine the model fit.

To address **Objective 6**, reproductive profile changes of women with PCOS will be compared with control subjects.

**Statistical analysis**

The distribution of variables will be examined using the Kolmogorov-Smirnov test. Difference between groups be evaluated using Student’s t-test for parametric data or Mann-Whiteney U test for nonparametric data as appropriate for continuous variables. To examine differences in frequencies and factors associated with incident diabetes, Chi-square or Fisher’s exact tests will be used as appropariate for categorical variables. Paired t-test and Wilcoxon test will be used to compare continuous variables between the baseline and follow-up visit. To evaluate the effect of PCOS on metabolic traits and androgen levels, analysis of covariance (ANCOVA) will be applied, including BMI as a covariate. Univariable and multivariable Cox proportional hazard regression analysis will be used to identify the independent predictors for diabetes or metabolic syndrome in women with PCOS. PCOS patients with these conditions at baseline will be excluded before performing the Cox model analysis. Covariates will be categorized or log-transformed if needed. We will enter age and body mass index in the model and use the forward stepwise algorithm to select a group of predictors for diabetes or metabolic syndrome in these women. A minimum events-to-variables ratio of 10 will be maintained in the multivariate modeling to avoid overfitting. SPSS version 20.0 will be used for all these data analyses and statistical significance will be defined as a two-sided p value <0.05.

**Sample size calculation**

Based on the rate of incident diabetes in our ongoing follow-up evaluation of control subjects (4.6% over 11.5years), and assuming a 2.5 fold increased risk of incident diabetes among women with PCOS, a sample size of 243 in each group will have 80% power to detect a significant difference at α= 0.05. Assuming a mean difference of 0.1mm in carotid IMT between the PCOS and control groups, with standard deviation of 0.1mm, a sample size of 38 each group will have 99% power to detect a significant difference at α=0.05, while a sample size of 50 each group will have 99% to detect a significant difference at α=0.01.

**References**

1. Chan JC, Malik V, Jia W, Kadowaki T, Yajnik CS, Yoon KH, Hu FB. Diabetes in Asia: epidemiology, risk factors, and pathophysiology. *Jama* 2009;**301**(20)**:**2129-40.

2. Xu Y, Wang L, He J, Bi Y, Li M, Wang T, Wang L, Jiang Y, Dai M, Lu J, Xu M, Li Y, Hu N, Li J, Mi S, Chen CS, Li G, Mu Y, Zhao J, Kong L, Chen J, Lai S, Wang W, Zhao W, Ning G. Prevalence and control of diabetes in Chinese adults. *Jama* 2013;**310**(9)**:**948-59.

3. Li H, Oldenburg B, Chamberlain C, O'Neil A, Xue B, Jolley D, Hall R, Dong Z, Guo Y. Diabetes prevalence and determinants in adults in China mainland from 2000 to 2010: A systematic review. *Diabetes Res Clin Pract* 2012.

4. Yang W, Lu J, Weng J, Jia W, Ji L, Xiao J, Shan Z, Liu J, Tian H, Ji Q, Zhu D, Ge J, Lin L, Chen L, Guo X, Zhao Z, Li Q, Zhou Z, Shan G, He J. Prevalence of diabetes among men and women in China. *N Engl J Med* 2010;**362**(12)**:**1090-101.

5. **Ma RC**, Lin X, Jia W. Causes of type 2 diabetes in China. *Lancet Diabetes Endocrinol* 2014.

6. Joham AE, Ranasinha S, Zoungas S, Moran L, Teede HJ. Gestational diabetes and type 2 diabetes in reproductive-aged women with polycystic ovary syndrome. *J Clin Endocrinol Metab* 2014;**99**(3)**:**E447-52.

7. **Ma RC**, Chan JC. Pregnancy and diabetes scenario around the world: China. *Int J Gynaecol Obstet* 2009;**104**(Suppl 1)**:**S42-5.

8. Azziz R, Woods KS, Reyna R, Key TJ, Knochenhauer ES, Yildiz BO. The prevalence and features of the polycystic ovary syndrome in an unselected population. *J Clin Endocrinol Metab* 2004;**89**(6)**:**2745-9.

9. Li R, Zhang Q, Yang D, Li S, Lu S, Wu X, Wei Z, Song X, Wang X, Fu S, Lin J, Zhu Y, Jiang Y, Feng HL, Qiao J. Prevalence of polycystic ovary syndrome in women in China: a large community-based study. *Hum Reprod* 2013;**28**(9)**:**2562-9.

10. Goodarzi MO, Dumesic DA, Chazenbalk G, Azziz R. Polycystic ovary syndrome: etiology, pathogenesis and diagnosis. *Nat Rev Endocrinol* 2011;**7**(4)**:**219-31.

11. Jayasena CN, Franks S. The management of patients with polycystic ovary syndrome. *Nat Rev Endocrinol* 2014;**10**(10)**:**624-636.

12. Dunaif A, Segal KR, Futterweit W, Dobrjansky A. Profound peripheral insulin resistance, independent of obesity, in polycystic ovary syndrome. *Diabetes* 1989;**38**(9)**:**1165-74.

13. Legro RS, Kunselman AR, Dodson WC, Dunaif A. Prevalence and predictors of risk for type 2 diabetes mellitus and impaired glucose tolerance in polycystic ovary syndrome: a prospective, controlled study in 254 affected women. *J Clin Endocrinol Metab* 1999;**84**(1)**:**165-9.

14. Ehrmann DA, Barnes RB, Rosenfield RL, Cavaghan MK, Imperial J. Prevalence of impaired glucose tolerance and diabetes in women with polycystic ovary syndrome. *Diabetes Care* 1999;**22**(1)**:**141-6.

15. Celik C, Tasdemir N, Abali R, Bastu E, Yilmaz M. Progression to impaired glucose tolerance or type 2 diabetes mellitus in polycystic ovary syndrome: a controlled follow-up study. *Fertil Steril* 2014;**101**(4)**:**1123-8 e1.

16. Gambineri A, Patton L, Altieri P, Pagotto U, Pizzi C, Manzoli L, Pasquali R. Polycystic ovary syndrome is a risk factor for type 2 diabetes: results from a long-term prospective study. *Diabetes* 2012;**61**(9)**:**2369-74.

17. Dunaif A. Insulin resistance and the polycystic ovary syndrome: mechanism and implications for pathogenesis. *Endocr Rev* 1997;**18**(6)**:**774-800.

18. Diamanti-Kandarakis E, Dunaif A. New perspectives in polycystic ovary syndrome. *Trends Endocrinol Metab* 1996;**7**(8)**:**267-71.

19. Talbott EO, Guzick DS, Sutton-Tyrrell K, McHugh-Pemu KP, Zborowski JV, Remsberg KE, Kuller LH. Evidence for association between polycystic ovary syndrome and premature carotid atherosclerosis in middle-aged women. *Arterioscler Thromb Vasc Biol* 2000;**20**(11)**:**2414-21.

20. Valkenburg O, Steegers-Theunissen RP, Smedts HP, Dallinga-Thie GM, Fauser BC, Westerveld EH, Laven JS. A more atherogenic serum lipoprotein profile is present in women with polycystic ovary syndrome: a case-control study. *J Clin Endocrinol Metab* 2008;**93**(2)**:**470-6.

21. Wild RA, Rizzo M, Clifton S, Carmina E. Lipid levels in polycystic ovary syndrome: systematic review and meta-analysis. *Fertil Steril* 2011;**95**(3)**:**1073-9 e1-11.

22. Yildiz BO, Bozdag G, Yapici Z, Esinler I, Yarali H. Prevalence, phenotype and cardiometabolic risk of polycystic ovary syndrome under different diagnostic criteria. *Hum Reprod* 2012;**27**(10)**:**3067-73.

23. Lam PM, Tam WH, **Cheung LP**. Higher metabolic risk in Chinese women fulfilling the NIH diagnostic criteria for polycystic ovarian syndrome. *Fertil Steril* 2009;**91**(4 Suppl)**:**1493-5.

24. Daan NM, Louwers YV, Koster MP, Eijkemans MJ, de Rijke YB, Lentjes EW, Fauser BC, Laven JS. Cardiovascular and metabolic profiles amongst different polycystic ovary syndrome phenotypes: who is really at risk? *Fertil Steril* 2014.

25. Azziz R, Carmina E, Dewailly D, Diamanti-Kandarakis E, Escobar-Morreale HF, Futterweit W, Janssen OE, Legro RS, Norman RJ, Taylor AE, Witchel SF. Positions statement: criteria for defining polycystic ovary syndrome as a predominantly hyperandrogenic syndrome: an Androgen Excess Society guideline. *J Clin Endocrinol Metab* 2006;**91**(11)**:**4237-45.

26. National Institutes of Health. Evidence-based methodology workshop on polycystic ovary syndrome: National Institutes of Health, 2014.

27. Fauser BC, Tarlatzis BC, Rebar RW, Legro RS, Balen AH, Lobo R, Carmina E, Chang J, Yildiz BO, Laven JS, Boivin J, Petraglia F, Wijeyeratne CN, Norman RJ, Dunaif A, Franks S, Wild RA, Dumesic D, Barnhart K. Consensus on women's health aspects of polycystic ovary syndrome (PCOS): the Amsterdam ESHRE/ASRM-Sponsored 3rd PCOS Consensus Workshop Group. *Fertil Steril* 2012;**97**(1)**:**28-38 e25.

28. **Cheung LP**, **Ma RC**, Lam PM, Lok IH, Haines CJ, So WY, Tong PC, Cockram CS, Chow CC, Goggins WB. Cardiovascular risks and metabolic syndrome in Hong Kong Chinese women with polycystic ovary syndrome. *Hum Reprod* 2008;**23**(6)**:**1431-8.

29. Liang SJ, Hsu CS, Tzeng CR, Chen CH, Hsu MI. Clinical and biochemical presentation of polycystic ovary syndrome in women between the ages of 20 and 40. *Hum Reprod* 2011;**26**(12)**:**3443-9.

30. Conway G, Dewailly D, Diamanti-Kandarakis E, Escobar-Morreale HF, Franks S, Gambineri A, Kelestimur F, Macut D, Micic D, Pasquali R, Pfeifer M, Pignatelli D, Pugeat M, Yildiz B. European survey of diagnosis and management of the polycystic ovary syndrome: results of the ESE PCOS Special Interest Group's Questionnaire. *Eur J Endocrinol* 2014;**171**(4)**:**489-98.

31. Carmina E. Polycystic ovary syndrome: metabolic consequences and long-term management. *Scand J Clin Lab Invest Suppl* 2014;**244:**23-6; discussion 26.

32. Schmidt J, Brannstrom M, Landin-Wilhelmsen K, Dahlgren E. Reproductive hormone levels and anthropometry in postmenopausal women with polycystic ovary syndrome (PCOS): a 21-year follow-up study of women diagnosed with PCOS around 50 years ago and their age-matched controls. *J Clin Endocrinol Metab* 2011;**96**(7)**:**2178-85.

33. Carmina E, Campagna AM, Lobo RA. A 20-year follow-up of young women with polycystic ovary syndrome. *Obstet Gynecol* 2012;**119**(2 Pt 1)**:**263-9.

34. Winters SJ, Talbott E, Guzick DS, Zborowski J, McHugh KP. Serum testosterone levels decrease in middle age in women with the polycystic ovary syndrome. *Fertil Steril* 2000;**73**(4)**:**724-9.

35. Ramachandran A, **Ma RC**, Snehalatha C. Diabetes in Asia. *Lancet* 2010;**375**(9712)**:**408-18.

36. Chan JC, Cockram CS. Epidemiology of Diabetes Mellitus in China. In: Ekoe J-M, Rewers M, Williams R, Zimmet P, eds. The Epidemiology of Diabetes Mellitus. Second ed. Chichester: John Wiley and Sons Ltd, 2008: 179-206.

37. **Ma RC**, Lin X, Jia W. Causes of Type 2 Diabetes in China. *Lancet Diabetes and Endocrinology* 2014, Sept 10.

38. **Ma RC**, Chan JC. Type 2 diabetes in East Asians: similarities and differences with populations in Europe and the United States. *Ann N Y Acad Sci* 2013;**1281:**64-91.

39. Luk AO, Lau ES, So WY, **Ma RC**, Kong AP, Ozaki R, Chow FC, Chan JC. Prospective study on the incidences of cardiovascular-renal complications in chinese patients with young-onset type 1 and type 2 diabetes. *Diabetes Care* 2014;**37**(1)**:**149-57.

40. Chan JC, Lau ES, Luk AO, Cheung KK, Kong AP, Yu LW, Choi KC, Chow FC, Ozaki R, Brown N, Yang X, Bennett PH, **Ma RC**, So WY. Premature Mortality and Co-morbidities in Young-onset Diabetes - A 7 year Prospective Analysis. *Am J Med* 2014.

41. Yeung RO, Zhang Y, Luk A, Yang W, Sobrepena L, Yoon KH, Aravind SR, Sheu W, Nguyen TK, Ozaki R, Deerochanawong C, Tsang CC, Chan WB, Hong EG, Do TQ, Cheung Y, Brown N, Goh SY, **Ma RC**, Mukhopadhyay M, Ojha AK, Chakraborty S, Kong AP, Lau W, Jia W, Li W, Guo X, Bian R, Weng J, Ji L, Rosa MR, Toledo RM, Himathongkam T, Yoo SJ, Chow CC, Ho LL, Chuang LM, Tutino G, Tong PC, So WY, Wolthers T, Ko G, Lyubomirsky G, Chan JC. Metabolic profiles and treatment gaps in young-onset type 2 diabetes in Asia (the JADE programme): a cross-sectional study of a prospective cohort. *Lancet Diabetes Endocrinol* 2014; Jul 28.

42. Tutino GE, Tam WH, Yang X, Chan JC, Lao TT, **Ma RC**. Diabetes and pregnancy: perspectives from Asia. *Diabet Med* 2014;**31**(3)**:**302-18.

43. **Ma RC**, Chan JC, Tam WH, Hanson MA, Gluckman PD. Gestational diabetes, maternal obesity, and the NCD burden. *Clin Obstet Gynecol* 2013;**56**(3)**:**633-41.

44. Grundy S, Cleeman J, Daniels S, Donato K, Eckel R, Franklin B, Gordon D, Krauss R, Savage P, Smith SJ, Spertus J, Costa F, American Heart Association; National Heart L, and Blood Institute,. Diagnosis and management of the metabolic syndrome: an American Heart Association/National Heart, Lung, and Blood Institute Scientific Statement. *Circulation* 2005;**112:**2735-52.

45. **Ma RC**, **Liu KH**, Lam EP, **Cheung LP**, So WY, Kong APS, Chow CC, Tong PC, Chan JC. Sonographic measurement of mesenteric fat identifies presence of metabolic syndrome among Chinese subjects with Polycystic Ovary Syndrome. Annual Meeting, The Endocrine Society. Toronto, Canada, 2007.

46. **Liu K**, Chan Y, Chan W, Kong W, Kong M, Chan J. Sonographic measurement of mesenteric fat thickness and its association with cardiovascular risk factors: comparison with subcutaneous and preperitoneal fat thickness, magnetic resonance imaging and anthropometric indexes. *International Journal of Obesity* 2003;**27:**1267-73.

47. **Liu KH**, Chan YL, Chan JCN, Chan WB. Association of carotid intima-media thickness with mesenteric, preperitoneal and subcutaneous fat thickness. *Atherosclerosis* 2005;**179:**299-304.

48. **Liu K**, Chan YL, Chan JC, Chan WB, Kong WL. Mesenteric fat thickness as an independent determinant of fatty liver. *International Journal of Obesity* 2006**:**Jan 17; [Epub ahead of print].

49. **Liu K**, Chan Y, Chan W, Chan J, Chu W. Mesenteric fat thickness is an independent determinant of metabolic syndrome and defines subjects with increased carotid intima-media thickness. *Diabetes Care* 2006;**29:**379–384.

50. **Ma RC**, **Liu KH**, Lam PM, **Cheung LP**, Tam WH, Ko GT, Chan MH, Ho CS, Lam CW, Chu WC, Tong PC, So WY, Chan JC, Chow CC. Sonographic measurement of mesenteric fat predicts presence of fatty liver among subjects with polycystic ovary syndrome. *J Clin Endocrinol Metab* 2011;**96**(3)**:**799-807.

51. **Ma RC**, **Liu KH**, Jiang GZ, Yau T, Luk AO, Lam PM, Chan MH, Ho CS, Lam CW, Kong APS, So WY, Chan JC, **Cheung LP**, Chu W, Chow CC, Tam WH. Increased mesenteric fat thickness and visceral adiposity in Chinese women with Polycystic Ovary Syndrome. The 16th International Congress of Endocrinology. Chicago, U.S.A., 2014.

52. Ko GT, So WY, Tong P, **Ma RC**, Kong AP, Ozaki R, Yang X, Ho CS, Lam CW, Chan JC. Hypoadiponectinaemia enhances waist circumference as a predictor of glucose intolerance and clustering of risk factors in Chinese men. *Diabetes Metab*.

53. **Ma RC**, Ng ACW, **Liu KH**, Ko GTC, So WY, Chan JCN, Chow CC. Association between serum omentin-1, visceral adiposity and cardiometabolic risk factors in Chinese adults. 9th IDF-WPR Congress and 4th Scientific Meeting of the Asian Association for the Study of Diabetes. Kyoto, Japan, 2012.

54. **Ma RC**, Tong PC, Chan JC, Chow CC. Increased visfatin in Chinese subjects with polycystic ovary syndrome. 3rd International Congress on Prediabetes and the Metabolic Syndrome. Nice, France, 2009.

55. Revised 2003 consensus on diagnostic criteria and long-term health risks related to polycystic ovary syndrome (PCOS). *Hum Reprod* 2004;**19**(1)**:**41-7.

56. Ko GT, Chan JC, Chan AW, Wong PT, Hui SS, Tong SD, Ng SM, Chow F, Chan CL. Association between sleeping hours, working hours and obesity in Hong Kong Chinese: the 'better health for better Hong Kong' health promotion campaign. *Int J Obes (Lond)* 2007;**31**(2)**:**254-60.

57. James PA, Oparil S, Carter BL, Cushman WC, Dennison-Himmelfarb C, Handler J, Lackland DT, LeFevre ML, MacKenzie TD, Ogedegbe O, Smith SC, Jr., Svetkey LP, Taler SJ, Townsend RR, Wright JT, Jr., Narva AS, Ortiz E. 2014 evidence-based guideline for the management of high blood pressure in adults: report from the panel members appointed to the Eighth Joint National Committee (JNC 8). *Jama* 2014;**311**(5)**:**507-20.
