# Supplementary material for: Progression of glucose intolerance and cardiometabolic risk factors over a decade in Chinese women with polycystic ovary syndrome: A case-control study
Source: PLoS Med. 2019 Oct 25;16(10):e1002953. doi: 10.1371/journal.pmed.1002953 (PMC6814217; doi:10.1371/journal.pmed.1002953)
Supplement: S2 Doc — (DOCX) [file pmed.1002953.s003.docx]

| Name: | **Date of FU today:** |
| --- | --- |
| HKID: | **Age as of today:** y.o |
| DOB: | **Date of Initial Assessment:** |
| Contact No: | **Age of Initial Assessment:** y.o |

| **PCOS Criteria Satisfied (2 out of 3 criteria)** |  |
| --- | --- |
| - Oligo-amenorrhoea or oligo- anovulation | No / Yes |
| - Clinical and /or biochemical signs of hyperandrogenism | No / Yes |
| - Polycystic ovaries on ultrasound examination | No / Yes / Unknown |
| **Age of Diagnosis of PCOS:** | y.o |

| **HISTORY** | | | | | | **At initial assessment** | | **As of today** |
| --- | --- | --- | --- | --- | --- | --- | --- | --- |
| **Menstrual History** | | | | | | | | |
| Menstrual cycle: | 1. Regular / 2. Irregular / 3. Menopause | | | | |  | |  |
| Cycle length: | Cycle length in natural cycle (___ to ___ D) OR  Average cycles/year (______ cycles/yr) | | | | |  | |  |
| Oligoamenorrhoea | 0. No / 1. Yes | | | | |  | |  |
| Tx for menstrual problem: | 0. No / 1. COC / 2. Progestogens / 3. Others (specify)  (please indicate current or previous Tx) | | | | |  | |  |
| Onset of oligoamenorrhoea and duration of symptoms (if applicable): Start at ______ y.o and last ________ years | | | | | |  | |  |
| Age of menopause (if applicable) : _____________ y.o. | | | | | |  | |  |
| **Androgen Excess** | | | | | | | | |
| Clinical acne | | 0. No / 1. Mild / 2. Moderate / 3. Severe | | | |  | |  |
| Clinical hirsutism | | 0. No / 1. Mild / 2. Moderate / 3. Severe | | | |  | |  |
| Ferriman-Gallwey Score (if applicable) | | | | | |  | |  |
| Any treatment required for clinical hyperandrogenism | | 0. No / 1. Hormonal Drug / 2. Non-hormonal Drug / 3. Other non-pharmacological Tx (please specify) | | | |  | |  |
| **Reproductive History:** | | | | | | | | |
| Obstetric history  (please specify number) | | a) Gravida:  b) Parity: | | | | G P | | G P |
| Past obstetric history:  (please specify number) | | a) Livebirth:  b) Spontaneous Miscarriage / Abortion:  c) Ectopic:  d) Molar:  e) TOP | | | | L  A  E  M  T | | L  A  E  M  T |
| GDM in previous pregnancy | | 0. No / 1. Yes with diet only / 2. Yes with insulin Tx | | | |  | |  |
| Hx of recurrent miscarriage: (>=3consecutive miscarriage) | | 0. No / 1. Yes (please describe in detail) | | | |  | |  |
| Hx of infertility | | 0. No / 1. Yes | | | |  | |  |
| Duration of infertility (if applicable): ________ years | | | | | |  | |  |
| Infertility causes: | | 0. No / 1. Anovulation / 2. Others: (please specify) | | | |  | |  |
| Infertility treatment | | 0. No / 1. Drug / 2. Surgery / 3. ART | | | |  | |  |
| Hx of taking metformin for anovulation / infertility | | 0. No / 1. Yes (please specify when and duration given) | | | |  | |  |
| Any symptom improvement after metformin (if applicable) | | 0. No / 1. Yes (please specify what kind of symptoms) | | | |  | |  |
| Hx of ovarian drilling for treatment of PCOS | | 0. No / 1. Yes (please specify when was the operation) | | | |  | |  |
| Any symptom improvement after ovarian drilling (if applicable) | | 0. No / 1. Yes (please specify what kind of symptoms) | | | |  | |  |
| **Medical History** | | | | | | | | |
| Known HT | | 0. No / 1. Yes  (specify age of diagnosis & treatment and duration given - Anti-HT drugs) | | | |  | |  |
| Known DM | | 0. No / 1. Yes  (specify age of diagnosis & treatment and duration given - Diet / OHA / Insulin) | | | |  | |  |
| Known dyslipidemia | | 0. No / 1. Yes  (specify age of diagnosis & treatment and duration given - Diet / Lipid-lowering drug) | | | |  | |  |
| Known CVD | | 0. No / 1. Yes  (specify age of diagnosis and type of CVD - Angina / MI / CHF/ CVA/ PVD/ OT) | | | |  | |  |
| Other diseases (e.g. endometrial hyperplasia or hormone dependent tumor) | | 0. No / 1. Yes  (specify the disease, age of diagnosis & treatment and duration given) | | | |  | |  |
| Other psychiatric diseases | | 0. No / 1. Yes  (specify the disease, age of diagnosis & treatment and duration given) | | | |  | |  |
| **Social & Family History** | | | | | | | | |
| Smoking | | 0. Never smoker / 1. Ex-smoker / 2. Active smoker  (please specify how much cig/day) | | | |  | |  |
| Alcohol | | 0. Never smoker / 1. Social drinker / 2. Regular drinker  (please specify how much units/wk) | | | |  | |  |
| Exercise | | 0. No / 1. Seldom / 2. Regular  (please specify how much hrs/wk) | | | |  | |  |
| Family Hx of DM | | Father N / Y | | | Mother N / Y | | Siblings N / Y ____ | |
| Family Hx (for female) of oligo-amenorrhoea / hirsutism / known PCOS: No / Yes  Mother ____ // Sisters _____ ( ) out of ( ) F siblings (not including self) // Unknown | | | | | | | | |
| Family Hx (for male) of premature balding before age 30: No / Yes  Father ____ // Brothers _____ ( ) out of ( ) M siblings // Unknown | | | | | | | | |
| **Anthropometric Measurements** | | | | | | **At initial assessment** | | **As of today** |
| Height (m) | | |  | | |  | |  |
| Weight (kg) | | |  | | |  | |  |
| BMI | | |  | | |  | |  |
| Waist circumference (cm) | | |  | | |  | |  |
| Hip circumference (cm) | | |  | | |  | |  |
| BP (sbp / dbp mmHg) | | |  | | |  | |  |
| **Ultrasound Assessment** | | | | | | **At initial assessment** | | **As of today** |
| Polycystic ovaries on US | | | 0. No (please specify Abdo scan or TVS) / 1. Yes (unilateral ovary) / 2. Yes (bilateral ovaries) | | |  | |  |
| Left ovary  - Antral Follicle Count (AFC)  - Ovarian Volume | | | | | |  | |  |
| Right ovary  - Antral Follicle Count (AFC)  - Ovarian Volume | | | | | |  | |  |
| **Endocrine / Biochemical Tests** | | | | | | **At initial assessment** | | **As of today** |
| D2/3 [FSH] (IU/L) | | | |  | |  | |  |
| D2/3 [LH] (IU/L) | | | |  | |  | |  |
| D2/3 [E2] (pmol/L) | | | |  | |  | |  |
| [Testosterone] (nmol/L) | | | |  | |  | |  |
| [Androstenedione] (nmol/L) | | | |  | |  | |  |
| [17-OH Progesterone]: | | | |  | |  | |  |
| [SHBG] if done | | | |  | |  | |  |
| [Prolactin] (mIU/L) | | | |  | |  | |  |
| TFT - STSH (mIU/L) | | | |  | |  | |  |
| LFT - Total ALP (IU/L) | | | |  | |  | |  |
| LFT - ALT /GPT (IU/L) | | | |  | |  | |  |
| Urate (nmol/L) | | | |  | |  | |  |
| Kisspeptin (pmol/L) | | | |  | |  | |  |
| **Metabolic Tests** | | | | | | **At initial assessment** | | **As of today** |
| [glucose]fasting (mmol/L) | | | |  | |  | |  |
| [glucose]2-hour (mmol/L) | | | |  | |  | |  |
| [insulin]fasting (mU/L) | | | |  | |  | |  |
| [Cholesterol]fasting (mmol/L) | | | |  | |  | |  |
| [HDL-C]fasting (mmol/L) | | | |  | |  | |  |
| [Triglyceride]fasting (mmol/L) | | | |  | |  | |  |
| [LDL-C]fasting (mmol/L) | | | |  | |  | |  |
| **Other Tests** | | | | | | | | |
| **Stored blood sample for future research (? AMH) send to O&G Prof Sahota’s laboratory per instructed**  **5 ml clotted blood (room temp if within an hour / or stored in refrigerator up to 24 hours)** | | | | | | | | |

1. **SUMMERY OF INITIAL ASSESSMENT**

**Date of Initial Assessment: _______________**

**Sub-phenotypes of PCOS on initial assessment:**

□ Androgen excess + ovulatory dysfunction (AE+ OD)

□ Androgen excess + polcystic ovarian morphology (AE + PCOM)

□ Ovulatory dysfunction + polycystic ovarian morphology (OD+ PCOM)

□ Androgen excess + ovulatory dysfunction + polycystic ovarian morphology (AE+ OD+ PCOM)

**Metabolic risk factors on initial assessment:**

- **Disturbances of glucose (ADA 2003/ WHO 2006) or insulin metabolism:**

| □ IFG (FPG ≥ 5.6 mmol/L) □ IFG (FPG ≥ 6.0 mmol//l) | □ IGT (2 hr glucose >7.8 mmol/L) |
| --- | --- |
| □ T2DM (FPG ≥ 7 mmol/L or 2 hr glucose ≥11.1 mmol/L) | □ Hyperinsulinaemia |

- **Dyslipidaemia:**

| □ Elevated Triglyceride | □ Reduced HDL-C |
| --- | --- |

- **Metabolic Syndrome (ATPIII 2005):**

| □ Waist >80 cm | □ BP >130/85 mmHg | □ FPG >5.6 mmol/L |
| --- | --- | --- |
| □ TG >1.7 mmol/L | □ HDL-C <1.3 mmol/L | □ Fulfilling 3 or more criteria |

**Other ultrasound measurements at baseline (Date: _________________):**

| Carotid IMT: | Right | Left | Mean |
| --- | --- | --- | --- |
|  | Bulb | Common Carotid | |

| Subcutaneous fat thickness: |  | Mesenteric fat thickness: |  |
| --- | --- | --- | --- |
| Pre-peritoneal fat thickness: |  | Fatty liver score: |  |

**Other results/ Remarks:**

**B. SUMMERY OF FOLLOW UP VISIT**

**Date of follow-up visit: _______________**

**Sub-phenotypes of PCOS on follow-up assessment:**

□ Androgen excess + ovulatory dysfunction (AE+ OD)

□ Androgen excess + polcystic ovarian morphology (AE + PCOM)

□ Ovulatory dysfunction + polycystic ovarian morphology (OD+ PCOM)

□ Androgen excess + ovulatory dysfunction + polycystic ovarian morphology (AE+ OD+ PCOM)

**Any change in medical conditions in interim:**

|  | **No / Yes** | **When** | **Year Old** |
| --- | --- | --- | --- |
| **Diagnosed T2 DM** |  |  |  |
| **Diagnosed hyperlipidaemia** |  |  |  |
| **Diagnosed hypertension** |  |  |  |
| **Developed CHD** |  |  |  |
| **Diagnosed sleep apnoea** |  |  |  |
| **Other conditions:** |  |  |  |

**Metabolic risk factors on follow-up assessment:**

- **Disturbances of glucose (ADA 2003/WHO 2006) or insulin metabolism:**

| □ IFG (FPG ≥ 5.6 mmol/L) □ IFG (FPG ≥ 6.0 mmol//l) | □ IGT (2 hr glucose >7.8 mmol/L) |
| --- | --- |
| □ T2DM (FPG ≥ 7 mmol/L or 2 hr glucose ≥11.1 mmol/L) | □ Hyperinsulinaemia |

- **Dyslipidaemia:**

| □ Elevated Triglyceride | □ Reduced HDL-C |
| --- | --- |

- **Metabolic Syndrome (ATPIII 2005):**

| □ Waist >80 cm | □ BP >130/85 mmHg | □ FPG >5.6 mmol/L |
| --- | --- | --- |
| □ TG >1.7 mmol/L | □ HDL-C <1.3 mmol/L | □ Fulfilling 3 or more criteria |

**Other ultrasound measurements at FU (Date:_______________)**

| Carotid IMT: | Rt CCA | Lt CCA | Mean |
| --- | --- | --- | --- |
|  | Rt bulb | Left bulb | Mean |

| Subcutaneous fat thickness: |  | Mesenteric fat thickness: |  |
| --- | --- | --- | --- |
| Pre-peritoneal fat thickness: |  | Fatty liver  (No / mild / moderate / severe): |  |
| Aortic stiffness: |  | Mean renal resistance index: |  |

**Additional Abdominal US measurements on ovaries (First day of LMP: ____________)**

| **Right** ovary (dominant follicle / corpus luteal cyst on scan) | **No / Yes** |
| --- | --- |
| - Ovarian Volume |  |
| - Antral Follicle Count (AFC) |  |
| **Left** ovary (dominant follicle / corpus luteal cyst on scan) | **No / Yes** |
| - Ovarian Volume |  |
| - Antral Follicle Count (AFC) |  |

***Note:**

**1. Acne Hx:**

0 = no acne

1 = mild acne on the face only and not distressing

2 = moderate acne on the face only plus feel distressing

3 = severe acne involving both face & trunk

**2. Hirsutism Hx:**

0 = no hirsutism

1 = mild hirsutism (need no or occasional shaving <1 time/wk)

2 = moderate hirsutism (need regular shaving >1/wk but not distressing)

1. = severe hirsutism (need regular shaving >1/wk plus feel distressing)

**3. Hirsutism score** (Total Ferriman-Gallwey Score)

0-4 score for each of 11 body parts (score 0 indicates absence of terminal hair)

| Upper lip | 1. A few hairs at outer margin        1. A small moustache at outer margin 2. A moustache extending halfway from outer margin 3. A moustache extending to midline |
| --- | --- |
| Chin | 1. A few scattered hairs 2. Scattered hairs with small concentrations   3-4 Complete cover (3. light & 4. heavy) |
| Chest | 1. Circumareolar hairs 2. With midline hair in addition 3. Fusion of these areas, with ¾ cover 4. Complete cover |
| Upper back | 1. A few scattered hairs 2. Rather more, still scattered   3-4 Complete cover (3. light & 4. heavy) |
| Lower back | 1. A sacral tuft of hair 2. With some lateral extension 3. ¾ cover 4. Complete cover |
| Upper abdomen | 1. A few midline hairs 2. Rather more, still midline   3-4 Complete cover (3. light & 4. heavy) |
| Lower abdomen | 1. A few midline hairs 2. A midline streak of hair 3. A midline band of hair 4. An inverted V-shaped growth |
| Arm | 1. Sparse growth affecting ≤ ¼ of limb surface 2. More than this, cover still incomplete   3-4 Complete cover (3. light & 4. heavy) |
| Forearm | 1-4 Complete cover of dorsal surface; 2 grades of light and 2 of heavy growth |
| Thigh | As for arm |
| Leg | As for arm |


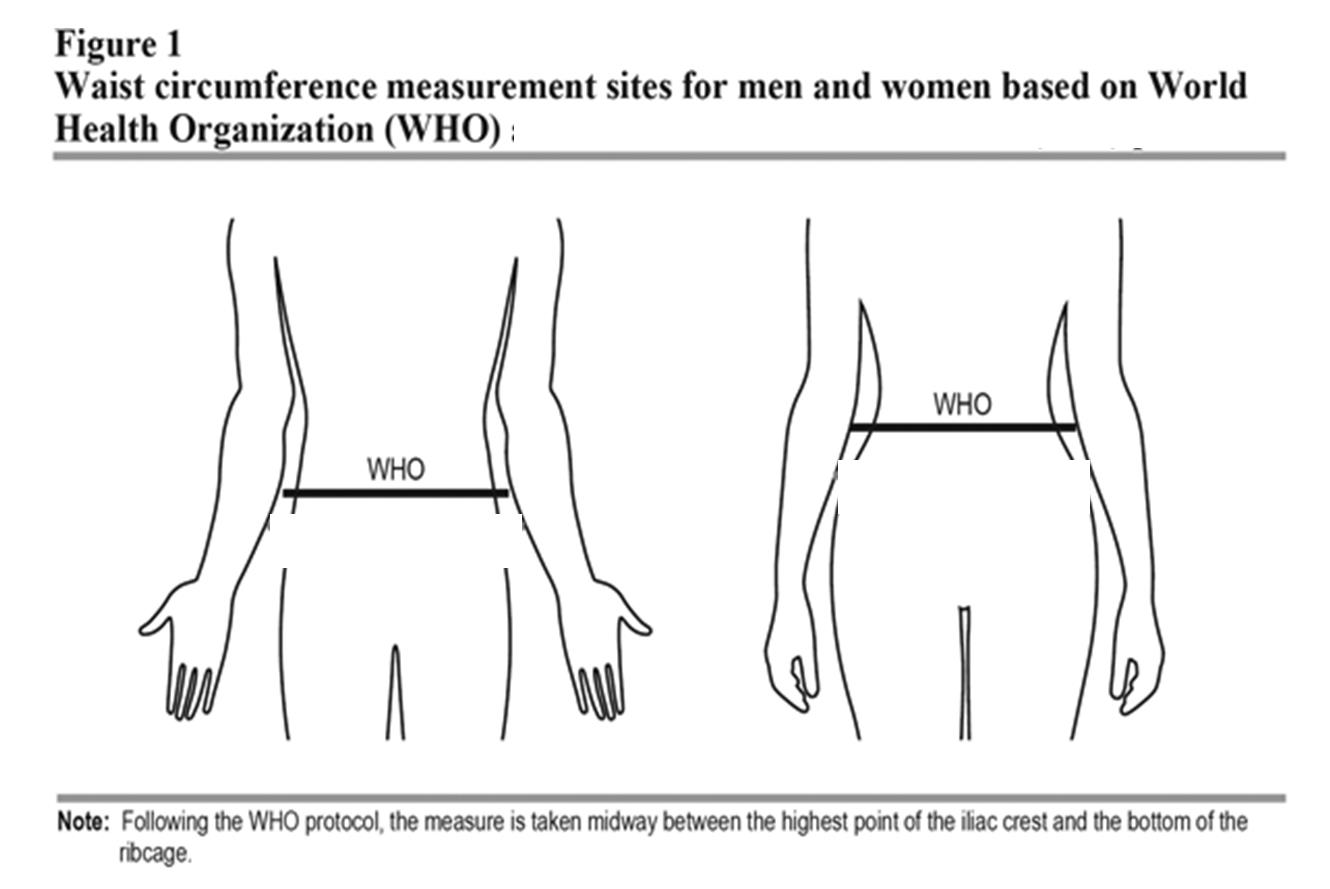


**4. Ultrasound appearance of polycystic ovary -** defined as the presence of 12 or more follicles in each ovary measuring 2-9 mm in diameter, and/or increased ovarian volume (>10ml).

- Follicle number is estimated in both longitudinal and antero-posterior cross-sections of the ovaries.
- The size of follicles is expressed as the mean of the diameters measured on the two sections.
- The ovarian volume is calculated by the simplified formula (0.5 x length x width x thickness).
- The pelvic USG is performed by trained medical staff, preferably via transvaginal approach.
- Oligo-/amenorrhoeic women are scanned at random time while regularly menstruating women will be scanned in the early follicular phase.
