# Supplementary figures and images for: Progression of glucose intolerance and cardiometabolic risk factors over a decade in Chinese women with polycystic ovary syndrome: A case-control study
Source: PLoS Med. 2019 Oct 25;16(10):e1002953. doi: 10.1371/journal.pmed.1002953 (PMC6814217; doi:10.1371/journal.pmed.1002953)

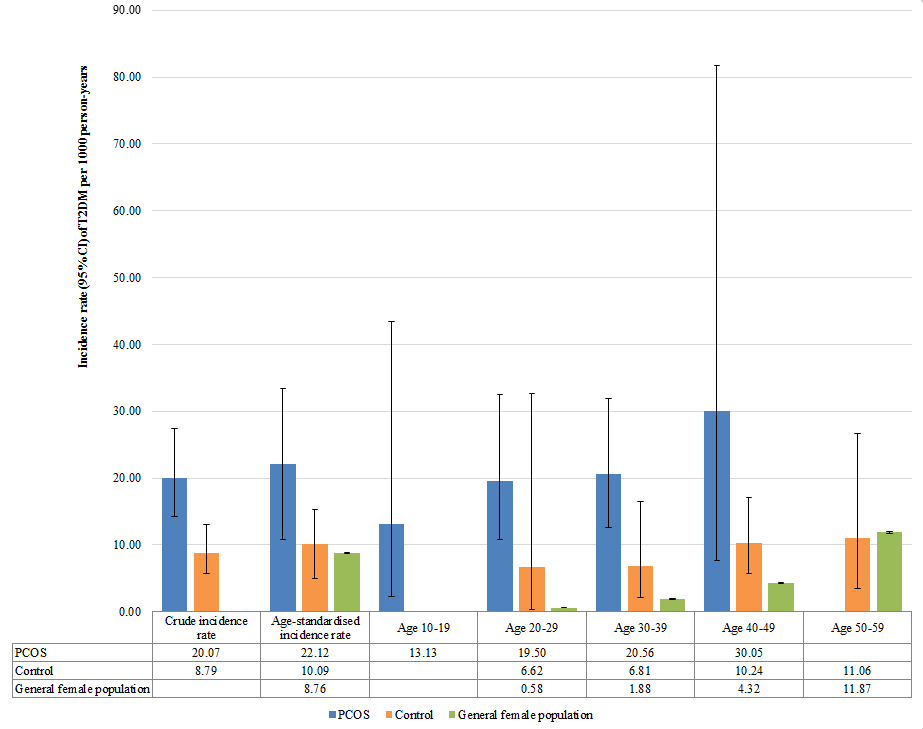

Supplement: S1 Fig — Glycaemic status was captured through 75-g OGTT in Chinese women with PCOS and women without PCOS, whereas that was ascertained through public sector electronic health records in the Hong Kong general female population. OGTT, oral glucose tolerance test; PCOS, polycystic ovary syndrome; T2DM, type 2 diabetes mellitus. (TIF) [file pmed.1002953.s008.tif]

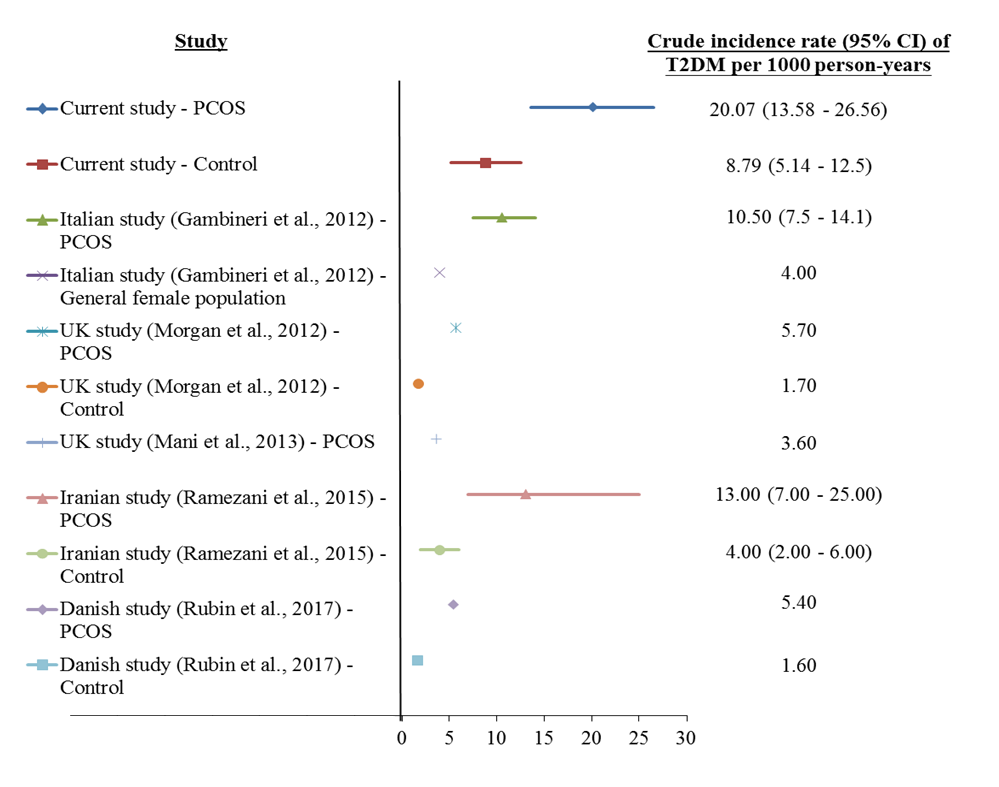

Supplement: S2 Fig — T2DM, type 2 diabetes mellitus. (TIF) [file pmed.1002953.s009.tif]
